# A long non-coding RNA interacts with Gfra1 and maintains survival of mouse spermatogonial stem cells

**DOI:** 10.1038/cddis.2016.24

**Published:** 2016-03-10

**Authors:** L Li, M Wang, M Wang, X Wu, L Geng, Y Xue, X Wei, Y Jia, X Wu

**Affiliations:** 1 State Key Laboratory of Reproductive Medicine (SKLRM), Nanjing Medical University, Nanjing, Jiangsu, China

## Abstract

Spermatogonial stem cells (SSCs) are unique male germline stem cells that support spermatogenesis and male fertility. Long non-coding RNAs (lncRNA) have been identified as key regulators of stem cell fate; however, their role in SSCs has not been explored. Here, we report that a novel spermatogonia-specific lncRNA (lncRNA033862) is essential for the survival of murine SSCs. LncRNA033862 is expressed in early spermatogonia including SSC and was among 805 lncRNAs identified by global expression profiling as responsive to glial cell-derived neurotrophic factor (GDNF), a growth factor required for SSC self-renewal and survival. LncRNA033862 is an antisense transcript of the GDNF receptor alpha1 (*Gfra1*) that lacks protein coding potential and regulates *Gfra1* expression levels by interacting with *Gfra1* chromatin. Importantly, lncRNA033862 knockdown severely impairs SSC survival and their capacity to repopulate recipient testes in a transplantation assay. Collectively, our data provide the first evidence that long non-coding RNAs (lncRNAs) regulate SSC fate.

Mammalian testes can produce thousands of sperm within the duration of a single heartbeat and millions of sperm per day. This output relies on spermatogenesis, a highly organized process encompassing proliferation and maturation of differentiating germ cells that ultimately derive from spermatogonial stem cells (SSCs). Surrounded by testicular somatic cells, SSCs represent male germline stem cells with the capability to develop into all types of male germline cells. Histological examination and transplantation experiments suggest that SSCs constitute a very rare cell population of the male gonad, encompassing approximately 0.01–0.03% of all cells in the mouse testis.^1, 2^ The maintenance of a SSC pool and balancing SSC self-renewal and differentiation are essential for life-long spermatogenesis and fertility.

With the development of protocols for the long-term propagation of mouse SSCs as clump-forming colonies *in vitro,*^3, 4^ multiple exogenous growth factors and chemicals have been identified that support the self-renewal and proliferation of mouse SSCs, including leukemia inhibitory factor,^3^ fibroblast growth factor 2 (FGF2),^3, 4^ epidermal growth factor,^4^ insulin growth factor 1 and colony-stimulating factor 1,^5^ Wnt glycoproteins^6^ and low-dose H_2_O_2,_^7^ among others. Survival and self-renewal mammalian SSC *in vivo* and *in vitro*^3, 4, 8^ is dependent on glial cell line-derived neurotrophic factor (GDNF), which is secreted by the testis niche. GDNF has been used *in vitro* to support maintenance and expansion of SSCs or SSC-like cells in many species from rodents to primate including human.^9, 10, 11, 12, 13^

Our understanding of molecular mechanisms controlling SSC fate has substantially increased within the past decade. GDNF signaling, alone or in combination with FGF2 (basic FGF, bFGF) signaling, predominantly acts through modification of the activity of protein kinases, which include phosphoinositide-3 kinase-AKT (PI3K-Akt), mitogen-activated protein kinase/ERK kinase and Src family kinases, and subsequent changes in phosphorylation of downstream substrates ultimately affect gene expression.^14, 15^ Additional evidence for factors involved in SSC self-renewal derives from experiments demonstrating that genetic activation of H-Ras, a member of the proto-oncogene Ras family, and of cell cycle protein cyclin D2 can support SSC-like cell proliferation *in vitro* without supplement of exogenous cytokines,^16^ and that activation of AKT allows for the long-term proliferation of SSCs in the presence of FGF2 and GDNF.^17^ In addition, a number of coding genes have been shown critical for SSC self-renewal and survival; these include both genes subject to regulation by exogenous growth factors, for example, *Etv5, Bcl6b*; and genes not subject to such regulation, for example, *Plzf, Foxo1.*^18, 19, 20, 21, 22^ Recently, it has also become evident that microRNAs, including miR21, miR20 and 106a, miR221 and 222, and let-7 family members have a role in maintaining a SSC pool and determining SSC fate.^23, 24, 25, 26^

LncRNAs are transcripts of >200 nucleotides in length with little or no potential for translation.^27^ Mammalian lncRNAs have been found to be intrinsically functional, and increasing evidence points toward a role of lncRNAs as determinants of stem cell fate; specifically, as regulators of potency, self-renewal and differentiation.^28, 29, 30^ Whether maintenance and differentiation of germline lineage stem cells depends on regulatory lncRNAs has not yet been investigated. Here, we have characterized lncRNA expression in SSCs by large-scale gene expression analysis, and report that a subset of lncRNA species in SSCs is regulated by GDNF, which is an essential growth factor required for SSC proliferation and maintenance. Among these, we have identified a novel spermatogonia-specific lncRNA (lncRNA033862) that controls SSC self-renewal and survival by regulation of the expression of *Gfrα1*, the glycosylphosphatidylinositol-linked cell surface receptor gene of GDNF.

## Results

### *In vitro* propagation of clump-forming germ cells with SSCs properties

**Figure 1:**
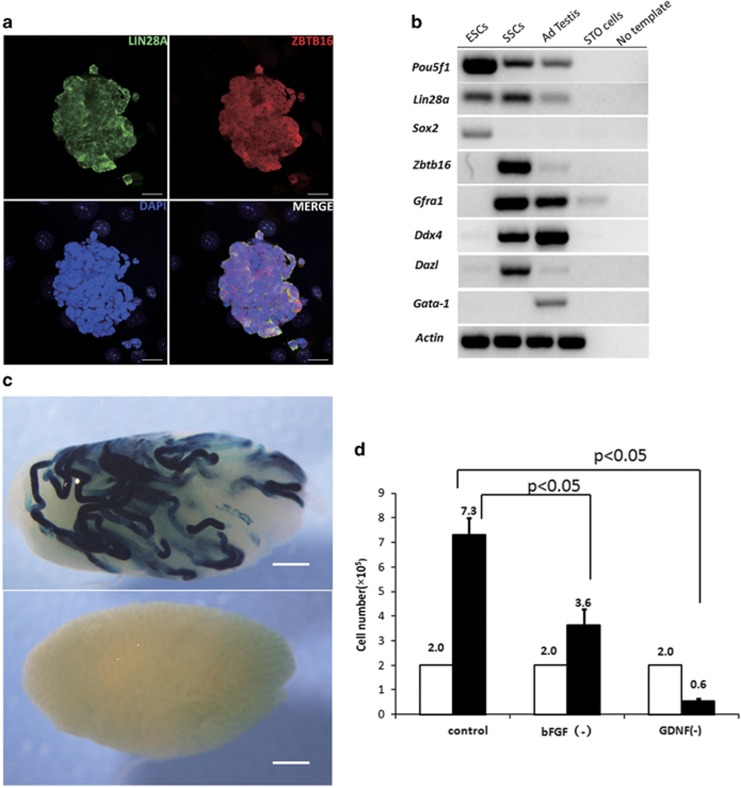
*In vitro* expansion of mouse SSCs. (**a**) SSC-enriched germ cells that were cultured in serum-free defined medium supplemented with GDNF and bFGF expanded into germ cell clumps that were positive for the pluripotency factor LIN28A, a cytoplasmic RNA binding protein, and the transcriptional factor ZBTB16, a marker for progenitor spermatogonia. Scale bar, 20 *μ*m. (**b**) Cultured SSCs exhibited a distinct expression profile of pluripotency genes and germ cell lineage-specific markers but not somatic cell markers expressed in testis (RT-PCR analysis). (**c**) SSCs cultured for 3 months *in vitro* efficiently repopulated germ cell-depleted recipient testes after transplantation, evident from the presence of beta-galactosidase-positive, donor-derived spermatogenic colonies (upper panel). Transplantation of vehicle medium only was used as negative control (lower panel), scale bar, 1 mm. (**d**) Proliferation of SSC in culture was dependent on GDNF but not bFGF. Cells plated at 2.0 × 10^5^ per well into culture medium without bFGF continued to proliferate and expanded (average 3.6 × 10^5^ per well *versus* 7.3 × 10^5^ per well in normal culture medium). Cells plated into culture medium lacking GDNF did not proliferate resulting in a net loss of cells (average 0.6 × 10^5^ per well) after 7 days culture. Control, normal culture medium contains both GDNF and bFGF. Error bars indicate S.D.

To establish SSC cultures, we isolated CD90.2 (Thy1)-positive germ cells from the testes of neonatal Rosa26 transgenic mice using magnetic cell separation. These cells were propagated *in vitro* for >3 months in a serum-free defined medium, with continuous replenishment of the growth factors GDNF, GFRA1 and FGF2, following a previously established and reproducible protocol.^31, 32^ The cultured germ cells formed clusters with typical grape-shaped morphology and were positive for PLZF (Zbtb16) and LIN28A (Figure 1a), which are markers of spermatogonial progenitor cells of the mouse testis.^20, 21, 33^ The expression profile of cultured germ cells was distinct from that of embryonic stem cells (ESCs) and STO feeder cells (feeder layer cells supporting ESC and SSC proliferation *in vitro*) and included pluripotency genes (*Pou5f1, Lin28a*), germline-specific genes (*Ddx4* and *Dazl*) and SSC markers (*Zbtb16* and *Gfra1*), but not *Gata1,* which is restricted to somatic lineages of the testis (Figure 1b). Cultures established from postnatal germ cells support the maintenance and expansion of SSCs but do not reflect a homogeneous stem cell population. Rather, similar to spermatogonia *in vivo*, these cultures contain a sub-population of true SSCs as defined by the capacity to restore spermatogenesis after transplantation into germ cell-depleted recipient mouse testes^34, 35^ We transplanted cell suspensions from SSCs cultures that had been maintained for 3 months into recipient testes and detected extensive colonization by beta-galactosidase reporter gene-positive donor cells (Figure 1c), confirming that the cultured clump-forming germ cells contained SSCs. Furthermore, *in vitro* proliferation of SSCs was dependent on GDNF: removal of GDNF from the culture medium for a period of 7 days resulted in a significant reduction in cell number (of 2.0 × 10^5^ plated cells, an average of 0.6 × 10^5^ remained after GDNF depletion) and changes in morphology with disappearance of grape shape clumps. In contrast, withdrawal of FGF2 for a week resulted in slower proliferation, but a net increase in cell number (2.0 × 10^5^ *versus* 3.6 × 10^5^), further confirming the crucial role of GDNF in maintaining SSC self-renewal and survival (Figure 1d and Supplementary Figure 1).

### Genome-wide sequencing identifies lncRNAs with a potential role in SSC self-renewal and survival

**Figure 2:**
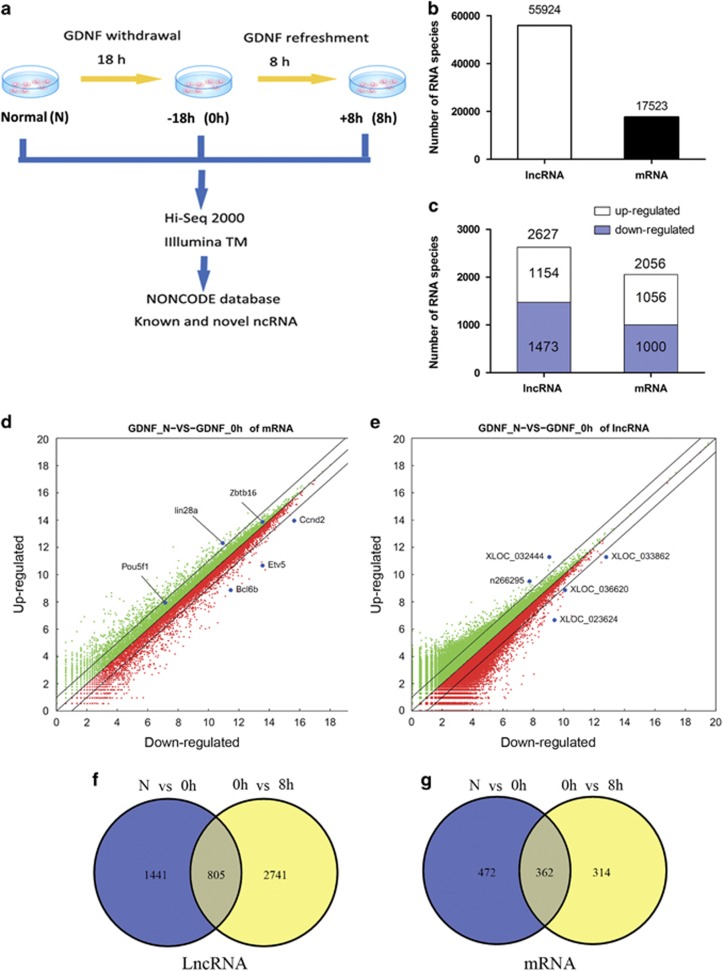
Global profiling of LncRNAs and mRNAs regulated by GDNF in SSCs. (**a**) Experimental design. SSCs from two independently established cultures were collected at three time points of GDNF exposure (N, in normal culture medium; 0 h, after 18 h of GDNF depletion; 8 h, after 8 h of replenishing GDNF). (**b**) Total number of lncRNA and mRNA species identified in SSCs. (**c**) Number of lncRNA and mRNA species that were significantly (at least twofold; *P*<0.05) upregulated (no fill) and downregulated (blue fill) in response to GDNF depletion. (**d** and **e**) Scatter plots of all lncRNAs and mRNAs identified in SSCs. A log2 calculation was used to normalize each transcript expression level. RNA species that were up- and downregulated after GDNF depletion are shown in red and green color, respectively. (**f**) Venn diagram analysis of lncRNA and mRNA species with significant (at least twofold; *P*<0.05) expression changes after GDNF withdrawal (N *versus* 0 h) and (**g**) replenishment (0 h *versus* 8 h). The number of lncRNA species with expression changes in response to GDNF withdrawal and replenishment was more than twofold higher than that of mRNA species (805 *versus* 362)

Omitting GDNF from the medium for up to 18 h does not adversely affect the SSC potential of cultured Thy1-positive germ cells; therefore, temporary GDNF removal and subsequent replenishment can be used to identify GDNF-regulated genes involved in SSC fate determination.^19^ We used high-throughput sequencing to compare global RNA transcript profiles of two independent SSC cultures harvested in three different culture conditions that included normal culture in GDNF and FGF2 supplemented medium, after 18 h of GDNF depletion, and after an additional 8-h period of re-exposure to GDNF (see flow diagram in Figure 2a). Overall, normal cultured SSCs (N) contained more than twice as many lncRNA species (55924) than mRNA transcripts (17 523); these included 21 949 known lncRNAs from the NONCODE library (version 3.0) and 33 975 predicted lncRNAs that were identified using Coding Potential Calculator^36^ (Figure 2b). Among mRNAs and lncRNAs with expression changes induced upon GDNF withdrawal (RNA transcripts with at least twofold up- or downregulation after 18 h of GDNF depletion compared with normal culture, *P*<0.05), comparable proportions exhibited increases and decreases in level (1473/1154 *versus* 1000/1056; Figure 2c), suggesting that mechanisms of GDNF-dependent up- and downregulation of transcription in the genome of mouse SSCs affect mRNAs and lncRNAs similarly. A similar regulation pattern was also found after GDNF refreshment (Supplementary Figure 2a). Among the mRNAs with expression changes in response to GDNF removal were several genes previously identified as responsive to GDNF signaling, including *Etv5*, *Bcl6b* and *Ccnd2* (Figure 2d). Known or predicted lncRNAs with expression changes after GDNF withdrawal included n266295, XLOC032444 and XLOC033862 (Figure 2e). For a more stringent analysis, we focused on transcripts that exhibited significant expression changes in response to both GDNF depletion and refreshment. A comparison of RNA species with at least a twofold change (mean reads from two replicates and Pearson correlation>0.99, *P*<0.05) in expression level between control (N), 18 h GDNF withdrawal (0 h) and 8-h GDNF re-exposure (8 h) identified 805 lncRNAs and 362 mRNAs (Figures 2f and g and Supplementary Figures 2b–d). These lncRNAs and mRNAs were randomly derived from all mouse chromosomes; most lncRNAs were either intergenic, intron antisense or intron sense (Supplementary Figures 3a and b). More stringent criteria (false detection rate, FDR<0.05) identified 83 lncRNAs including XLOC033862 that exhibited distinct expression patterns following both GDNF withdrawal and refreshment (Supplementary Figure 3c and Supplementary Table 1).

### LncRNA033862 is predominantly expressed in mouse SSCs and subject to GDNF signaling

**Figure 3:**
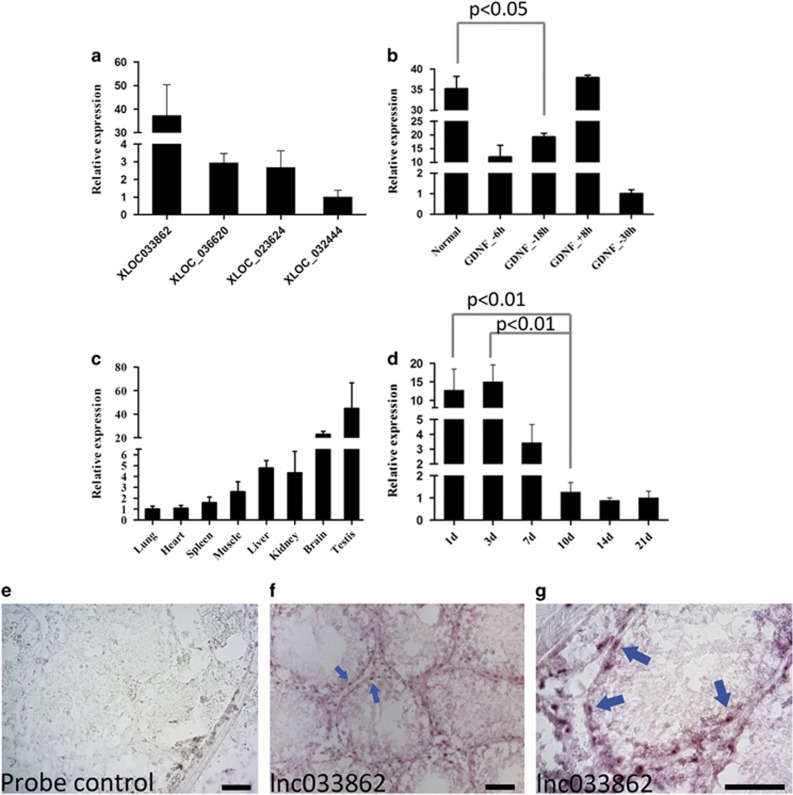
LncRNA033862 is highly expressed in cultured SSCs and in spermatogonia. Quantitative real-time RT-PCR confirmed (**a**) higher relative expression of lncRNA033862 in SSC compared with other predicted lncRNAs (lnc036620, lnc023642 and lnc0322444) that exhibited expression changes in response to GDNF withdrawal. (**b**) Changes in relative expression of lncRNA033862 after GDNF withdrawal and replenishment. (**c**) Tissue-specific expression analysis reveals high expression levels of lncRNA033862 in adult mouse testis and brain tissue. (**d**) Changes in lncRNA033862 transcript levels in mouse testis during the first 3 weeks of postnatal development; transcript levels were highest from postnatal day (P) 1 to P7 and significantly decreased after P10 (*P*-value <0.01). (**e**) *In situ* hybridization identifies cell type-specific expression of lncRNA033862 in spermatogonia on the basal membrane of seminiferous tubules, and the complementary sequence to the probe was used as sense control. (**f**) Predominantly nuclear signal was found at lower magnification and (**g**) higher magnification. Error bars indicate S.D. Scale bar=50 *μ*m

Among the 805 lncRNAs with significant expression changes in response to GDNF withdrawal, lncRNA033862 (mus-XLOC_033862, termed lncRNA033862), a predicted lncRNA of 6384 bp that was not in the NONCODE ncRNA V3.0 library but predicted by Coding Potential Calculator,^36^ had the highest number of sequencing reads; this lncRNA ranked 13th in the number of sequencing reads among all lncRNAs identified in SSCs (Supplementary Table 1). We used Cufflinks to assemble transcripts from all data sets using the RefSeq gene annotation as a reference guide,^37^ and found that LncRNA033862 is transcribed from mouse chromosome 19 in antisense direction of the GDNF family receptor alpha (*Gfra1*) gene and contains a single predicted exon (Supplementary Figures 4a and b). Using real-time reverse transcriptase polymerase chain reaction (RT-PCR; Supplementary Figure 4c), we confirmed that the relative expression level of lncRNA033862 was higher than that of other presumptive GDNF-regulated lncRNAs (036620, 023624, 032444; Figure 3a) and underwent significant changes in response to GDNF withdrawal (normal *versus* GDNF_–18 h, *P*<0.01) and replenishment (GDNF_–18 h *versus* GDNF_+8 h, *P*<0.01), with a decrease by almost 97% after 30 h of GDNF withdrawal (Figure 3b). Tissue-specific expression analysis revealed that lncRNA033862 was highly abundant in mouse testis and brain (Figure 3c). LncRNA033862 expression in postnatal mouse testis was stage specific; high levels of transcript were present during the immediate postnatal period (postnatal days (P) 1–3), a lower transcript level was detected at P7, and significantly lower levels after P10 (*P*<0.01,), suggesting that lncRNA033862 was predominantly expressed by gonocytes or progenitor spermatogonia, which represent the earlier undifferentiated germ cell population in mouse testis from P1 to P7 (Figure 3d). To assess cell type-specific expression patterns of lncRNA033862 in mouse testis, we performed *in situ* hybridization of testis cryosections from 5-month-old adult mice using a 794-bp RNA probe specific for lncRNA033862 (Supplementary Figure 4c). Hybridization signal was restricted to spermatogonial cells attached to the basal membrane of seminiferous tubules (Figures 3e–g and Supplementary Figure 5), suggesting that lncRNA033862 could be a spermatogonia-specific lncRNA.

### LncRNA033862 localizes to the nucleus and lacks translation potential

**Figure 4:**
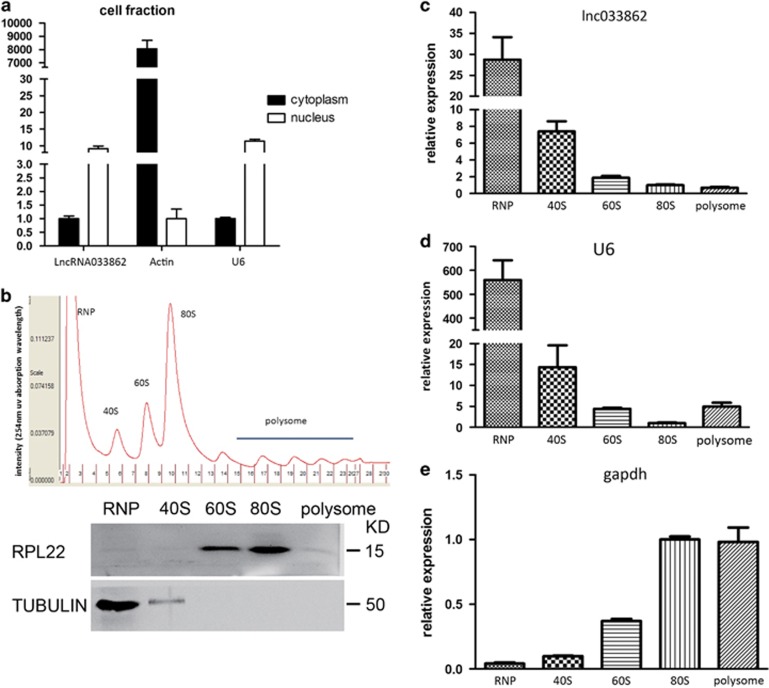
LncRNA 033862 localizes to the nucleus in SSCs and does not have translational potential. (**a**) Quantitative RT-PCR confirms that lncRNA033862 is predominantly found in the nuclear but not cytoplasmic fraction of SSCs. Small nuclear RNA U6 transcript and actin mRNA served as positive controls for a nuclear RNA and a translated mRNA, respectively. (**b**) Polysome profile of fractions from SSC. UV-absorbance peaks correspond to ribonucleoprotein particles (RNP), free ribosomal subunits (40S and 60S), 80S monosomes, and polysomes, separation of polysome fractions was confirmed by western blot analysis of ribosomal protein L22 (RPL22; positive control for polysome protein) and tubulin (positive control for RNP). (**c–e**) Quantitative real-time RT-PCR confirms the association of LncRNA033862 with RNP but not ribosomal subunits or monosomes. Small nucleus RNA U6 and GAPDH mRNA served as positive control for RNA species known to associate with RNP and ribosomes, respectively. Error bars indicate S.D.

To identify the subcellular localization of lncRNA033862, we isolated nuclear and cytoplasmic RNA species from SSCs after cellular fractionation (Supplementary Figure 7a). Similar to the small nuclear RNA U6, lncRNA033862 was abundant in the nuclear fraction of SSCs (Figure 4a). We next examined the coding potential of lncRNA033862, which was identified as a non-coding sequence using Coding Potential Calculator.^36^ Generally, it is believed that translation is actively associated with polysomes.^38, 39^ Therefore, we performed cell polysome separation and harvested RNAs from ribonucleoprotein (RNP), 40 s to 80 s ribosome, and polysome fractions isolated from SSCs by gradient centrifugation and fractionation (Figure 4b). Western blot analysis confirmed the absence of tubulin and ribosomal protein L22 (RPL22) from the polysome and RNP fractions, respectively (Figure 4b), validating separation efficiency. Amplification of transcripts associated with each polysomal fraction revealed that lncRNA033862 transcript was predominantly associated with RNPs but not ribosomes or polsysomes (Figure 4c), resembling the known nuclear transcript small nuclear RNA U6 in distribution (Figure 4d). In contrast, Gapdh mRNA, which served as a positive control for a translated transcript, was almost absent from the RNP fraction and associated with ribosome and polysome fractions (Figure 4e). Based on these findings, we propose that lncRNA033862 is an untranslated transcript without protein coding potential.

### LncRNA033862 is an antisense transcriptional regulator of *Gfra1*

**Figure 5:**
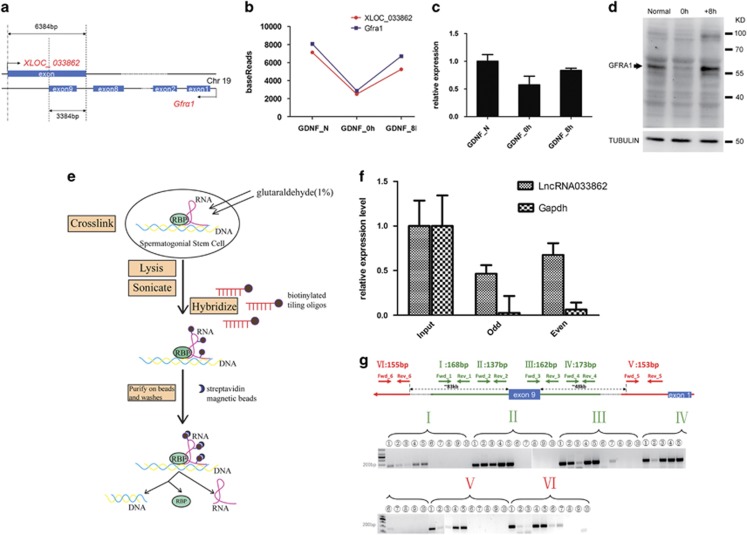
LncRNA033862 interacts with *Gfrα1* (**a**) LncRNA033862 is an antisense lncRNA of *Gfra1*, which encodes GDNF family receptor alpha. The 6384-bp sequence of lncRNA033862 is transcribed from the opposite strand of *Gfra1* covering intron 9 and exon 9. (**b**) Changes in lncRNA033862 and *Gfra1* transcript levels in SSC in response to GDNF depletion and replenishment. Y axis, number of reads identified by global gene expression profiling. (**c–d**) *Gfrα1* transcript and protein levels were further validated by RT-PCR and western blotting, respectively. (**e**) Experimental outline for ChIRP analysis. (**f**) ChIRP analysis demonstrates that lncRNA033862 interacts with the chromosomal region encoding *Gfra1* in SSCs. Biotinylated oligonucleotides (probes, total number=10) tiling the lncRNA033862 transcript were combined in even and odd-numbered pools. Each oligonucleotide pool specifically precipitated the lncRNA033862 transcript but not GAPDH mRNA transcript. (**g**) Five sets of PCR primers were designed to amplify those *Gfra1* DNA sequences that co-precipitated with the tiling oligonucleotides. At least one pool was enriched for all *Gfra1* sequences contained within the overlapping regions with lncRNA033862, but not sequences outside the region of complementarity. I and II: PCR primers directed against putative lncRNA033862 binding sequences located 2.3 and 0.6-kb downstream of exon 9 of the *Gfra1* gene, respectively; III and IV: primers detecting a sequence covering exon 9 of *Gfra1*; V: primers detecting a sequence at approximately 48-kb upstream of *Gfrα1* gene; VI: primers detecting a sequence at approximately 83-kb downstream of *Gfrα1* gene. PCR products were amplified using the respective primers indicated on top of the panel from the following samples: (1) input DNA, (2) and (3) DNA co-precipitated with odd and even-numbered pools of probes, (4) and (5) cell lysates, (6) to (9) washing buffers, and (10) water control. Error bars indicate S.D. for above statistic data

LncRNA033862 maps to *Gfra1*, which encodes the GDNF co-receptor expressed in SSCs.^40^ Specifically, lncRNA033862 is a partial antisense transcript of *Gfra1* that initiates 3-kb downstream of *Gfra1* exon 9 and includes exon 9 (2935 bp) and the intronic region between exons 8 and 9 (449 bp; Figure 5a; Supplementary Figures 4a and b). Global transcript analysis by RNA sequencing revealed that mRNA levels of *Gfra1* paralleled those of lncRNA033862 after GDNF removal and replenishment, with a decline in reads after 18 h of GDNF depletion and increase after GDNF replenishment (Figure 5b and Supplementary Figure 6a); these changes in *Gfra1* expression were confirmed on both the mRNA and protein levels (Figures 5c and d). Furthermore, there was a significant correlation (*P*<0.05) of *Gfra1* and lncRNA033862 expression *in vivo* (Supplementary Figure 6b).

It has been demonstrated that naturally occurring nuclear antisense transcripts can regulate gene expression by RNA–DNA interaction or RNA–protein interaction.^27^ To explore whether lncRNA033862 was potentially associated with and involved in *Gfra1* chromatin modification, we performed chromatin isolation by RNA purification (ChIRP; see flow diagram in Figure 5e). Cross-linked RNP–chromatin complexes were isolated after hybridization to tiled labeled antisense oligonucleotides designed against lncRNA033862 (Supplementary Figure 4c), followed by detection of captured RNA species and chromosomal segments. RT-PCR analysis of RNA species captured using two pools of five antisense oligonucleotides each revealed enrichment of lncRNA033862, whereas a control *Gapdh* mRNA was not captured (Figure 5f). We next amplified genomic DNA captured by ChIRP using oligonucleotides directed against regions of *Gfra1* that overlapped with or excluded the sequence of lncRNA033862. Significantly, at least in one set of tiling antisense oligo-captured DNAs, the regions of *Gfra1* gene overlapping with lncRNA033862 were much more enriched than non-overlapping regions (Figure 5g; this was further verified by quantitative PCR, Supplementary Figure 8a), suggesting that lncRNA033862 transcripts interact with *Gfra1* at regions of complementarity. We also evaluated potential interactions of lncRNA033862 with proteins by performing protein mass spectrometry on both sets of oligo-captured cross-linked RNP–chromatin complexes. These analyses identified only a single secretory carrier membrane protein SCAMP1 (Supplementary Figures 8b and c).

### Knockdown of lncRNA033862 impairs self-renewal and survival of mouse SSCs *in vitro*

**Figure 6:**
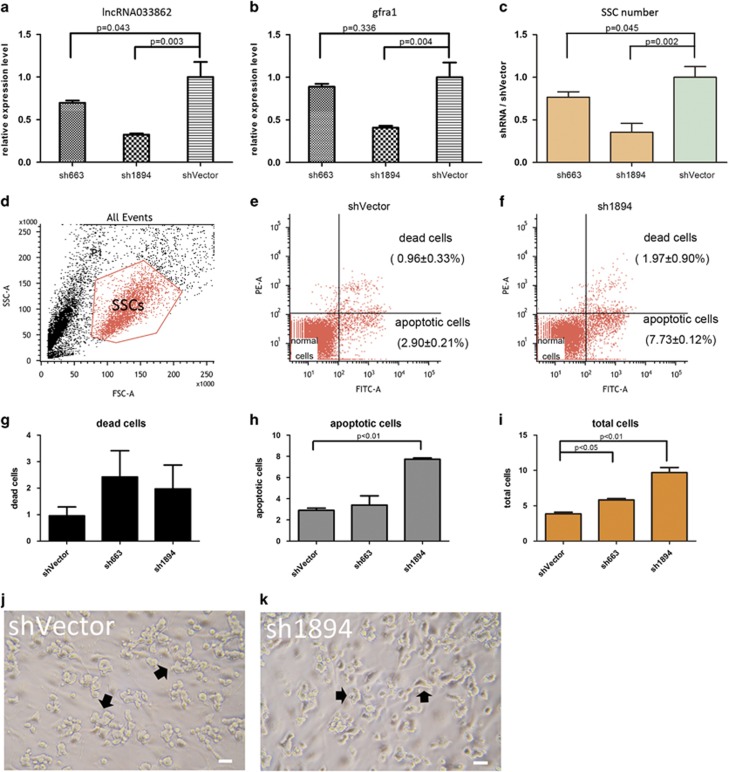
Knockdown of LncRNA 033862 impairs the survival and proliferation of SSCs**. (a)** *In vitro* lentivirus-mediated knockdown resulted in a reduction of lncRNA033862 transcript levels by 30% with shRNA663, and by 65% by shRNA1894, and a corresponding reduction of *Gfra1* transcript levels (**b**) to 88.9 % with shRNA663, and to 40.9% with shRNA1894. (**c**) Significant reduction of total cell number in SSC cultures 7 days after transduction with shRNA663 and shRNA1894 compared with vector alone (shVector). FACS analysis of SSCs gated according to forward and side scatter as indicated in (**d**). (**e** and **f**) Compared with shVector transfection, knockdown lncRNA033862 by sh1894 resulted in an increase of apoptotic cells in SSC cultures. (**g**) Lentiviral transduction of SSCs with shRNA663 or shRNA1894 resulted in an increased number of dead cells and (**h**) significantly higher total numbers of apoptotic cells in SSCs transduced with shRNA1894. (**i**) Both shRNA663 (*P*<0.05) and shRNA1894 (*P*<0.01) induced a significant increase in the number of abnormal (dead and apoptotic) cells. (**j**) Morphological changes in SSC cultures after vector control transfection and (**k**) shRNA1894 induced knockdown of lncRNA033862. Cell clumps became visibly smaller and dissociated within one week following transduction (scale bar=20 *μ*m). Error bars indicate S.D. for above data

To assess the effect of lncRNA033862 knockdown in SSCs on *Gfra1* expression and cell fate, we designed two different small hairpin RNAs (shRNAs) against lncRNA033862; both sh663 and sh1894 were directed against sequences of lncRNA033862 not contained within the presumptive region of interaction between lncRNA033862 and *Gfrα1* (Supplementary Figure 4c). Transduction of cultured SSCs with a lentivirus-encoding sh663 resulted in a reduction of lncRNA033862 transcript by 30% and a modest reduction of *Gfra1* expression levels (*P*>0.05). In contrast, transduction with a lentivirus-encoding sh1894 produced a significant reduction of lncRNA033862 transcript levels by 65% (*P*<0.05) in SSC cultures, indicating that this construct mediates effective knockdown of lncRNA033862, taking into account that not all cells were transduced. In these cultures, we observed a concomitant significant reduction of *Gfra1* mRNA levels by 60% (*P*<0.01; Figures 6a and b) and reduced GFRA1 protein levels (Supplementary Figure 7b). These results show that lncRNA033862 is a positive regulator of *Gfra1* transcription in SSCs.

After 7 days of culture, SSC cultures transduced with lentiviruses encoding sh663 or sh1894 had significantly lower cell numbers than control cultures (76.7% and 35.6% of control cell numbers, respectively; Figure 6c). FACS analysis revealed a larger proportion of apoptotic cells compared with control cultures (Figures 6d–f), with a significant increase of apoptotic cells and combined dead and apoptotic cells (Figures 6g–i, Supplementary Figure 7c) 4 days after lentiviral transduction. Cell cultures transduced with shRNA1894-encoding lentivirus underwent substantial changes in morphology, manifesting with an initial reduction of colony size followed by disappearance of grape-shaped clusters (Figures 6j and k), and a significant decrease in total cell number compared with control cultures (Figure 6c). Furthermore, we found that transcripts of genes associated with SSC self-renewal, including *Bcl6b Ccnd2*, and *Pou5f1* (*Oct4*), were significantly downregulated, whereas transcript levels of genes associated with differentiation, such as *Stra8*, *Sypc1* and *Kit*, were extremely low and not affected by lncRNA033862 knockdown; transcripts of genes associated with homing were unchanged (*beta1-integrin*) or reduced (*Cxcr4*; Supplementary Figure 9). In summary, these data suggest that knockdown of lncRNA033862 impairs self-renewal and maintenance of SSCs.

### Knockdown of lncRNA033862 impairs the capacity of SSC to regenerate spermatogenesis after transplantation

**Figure 7:**
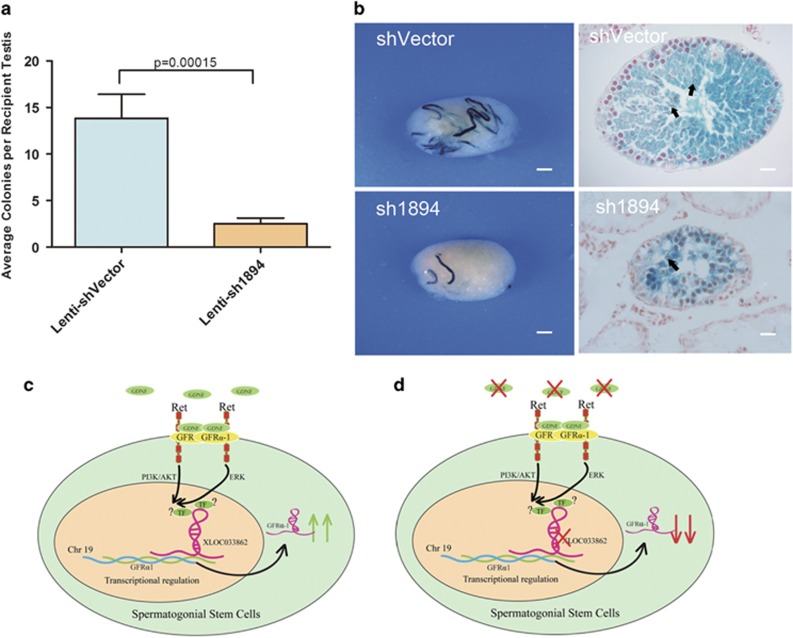
Reduced repopulation capacity of SSCs after lncRNA033862 knockdown. (**a** and **b**) SSCs transduced with shRNA1894-encoding lentivirus formed significantly fewer spermatogenic colonies in recipient testes than SSCs transduced with vector control, scale bar =1mm. Data are from three independent transplantation experiments; in total, control vector lentivirus transduced SSC were transplanted into 21 testes, and sh1894 lentivirus transduced SSC into 22 testes. Donor-derived colonies exhibited a full range of germ cell development, including mature spermatids (black arrows, scale bar=1 mm for left panel and 20 *μ*m for right panel), irrespective of lncRNA033862 knockdown. (**c**) Proposed model for the regulation of mouse SSC self-renewal and maintenance by lncRNA033862. Under normal physiologic conditions in the testes, exogenous growth factor GDNF binds to its receptor GFRA1 and RET and stimulates intracellular signaling involving PI3K/AKT and ETK pathways. This positively regulates transcription of lncRNA033862, such that sufficient transcript levels are present in the nucleus to activate the transcription of genes necessary for SSC self-renewal, such as *Gfra1*. (**d**) In the absence of GDNF, lower lncRNA033862 transcription, leading to reduced activation of *Gfra1* transcription, causes imbalance of self-renewal and maintenance of SSCs. Error bars indicate S.D.

SSC cultures contain a sub-population of true SSCs that are defined by their capacity to restore spermatogenesis in germ cell-depleted recipient testes after transplantation.^35^ To assess if lncRNA033862 knockdown affected properties of this sub-population, we performed transplantation assays into recipient testes.^41^ We transplanted cell suspensions of SSC cultures, in which overall transcript levels of lncRNA033862 were reduced up to 65% after transduction with a lentivirus-encoding sh1894, into the testes of recipient male mice in which endogenous spermatogenesis had been abolished. We observed significantly fewer donor SSC-derived germ cell colonies in testes that had received transplants from SSC cultures subject to lncRNA033862 knockdown compared with testes receiving cells from SSC cultures transduced with control lentivirus (2.5±0.6 *versus* 13.8±2.6, *P*<0.01; Figure 7a). Histological analysis showed that seminiferous tubules with donor-derived colonies from either lncRNA033862 knockdown or control SSC cultures exhibited a full range of germ cell development (Figure 7b). These findings demonstrate that knockdown of lncRNA033862 in SSC cultures via lentiviral transduction significantly reduces the number of true SSCs in these cultures; however, a subset of SSCs remain that are capable of restoring the full range of spermatogenesis. These may reflect cells that were not transduced.

In summary, we have identified the first lncRNA transcript that is important for the self-renewal function of SSCs. We propose that transcription of *Gfra1* requires a normal level of lncRNA033862, which is present under physiological conditions in the testis niche and depends on GDNF signaling (Figure 7c). The absence of GDNF from niche or culture causes a reduction in lncRNA033862 transcript level, resulting in transcriptional silencing of *Gfra1* (Figure 7d).

## Discussion

Growing evidence has revealed a major involvement of lncRNAs in stem cell fate determination, including maintaining self-renewal of stem cells and lineage differentiation.^28, 29, 30^ Here, we have applied high-throughput RNA sequencing to identify lncRNAs with a potential regulatory role in SSCs. Of 55 924 lncRNA transcripts, we identified 805 lncRNA transcripts in SSCs subject to transcript level changes in response to GDNF, which is an essential growth factor required for SSC self-renewal. These represent approximately 1.4% of the total sequenced known and predicted lncRNAs, suggesting a potential biological significance of these lncRNAs in GDNF-dependent self-renewal of SSCs.

To promote spermatogonial self-renewal, GDNF signals through a receptor complex that includes GDNF family receptor α1 (GFRA1). GFRA1 has been considered a cell surface marker suitable for the identification of SSCs in mouse testes. GFRA1-positive single spermatogonia have been considered the ‘actual stem cells' of the testes that have the greatest potential to support normal spermatogenesis,^42^ and SSCs have been successfully enriched using cell sorting with an anti-GFRA1 antibody.^43^ Genetic loss of *Gfra1* significantly reduced the self-renewal of SSCs in neonatal mouse testis,^44^ and *in vitro* reduction of *Gfra1* transcript negatively affected the proliferation of SSCs.^40^ Results of previous studies have suggested a feed-forward mechanism in which expression of the co-receptor Gfra1 is upregulated by GDNF signaling but the regulatory mechanism remains undefined. Although a mounting body of evidence indicates that lncRNAs influence the fate decisions of stem cells in other lineages, their role in any spermatogonial subtype has remained undefined. Here, we have identified lncRNA033862, a GDNF-regulated antisense lncRNA transcript located within *Gfra1* that is predominantly expressed in spermatogonia including SSCs of the mouse testis. We find that lncRNA033862 regulates SSC survival and proliferation by RNA–DNA interaction and mediates transcriptional activation of *Gfra1*, the receptor gene of GDNF.

Nuclear lncRNAs encompass natural antisense transcripts, defined as RNA molecules that are transcribed from the opposite DNA strand of a transcript and overlap in part with the sense RNA.^27^ The majority of natural antisense transcripts that have been identified in the mammalian genome are non-protein-coding.^27^ We find that lncRNA033862 represents such a nuclear, non-coding antisense transcript of *Gfra1*. Although many studies have demonstrated that antisense transcripts mediate both transcriptional activation or silencing of the associated coding gene,^45, 46, 47, 48, 49^ the mechanisms by which lncRNAs exert this regulatory control on gene expression remain elusive. Presumably, lncRNAs may affect gene expression and protein translation by interacting with RNAs transcripts, miRNAs or protein components of RNA-associated RNP complexes.^50^ In this study, we did not evaluate potential interactions of lncRNA033862 with miRNAs because of the exclusively nuclear localization of lncRNA033862, which would render such an association unlikely. We did not find significant protein partners of lncRNA033862 except for SCAMP1, which is a secretory carrier membrane protein located in the cell membrane.^51^ Based on these findings, we hypothesize that the positive effect of lncRNA033862 on *Gfra1* transcription is mediated via RNA–chromatin interactions. Potential RNA–DNA interaction affecting gene transcription have also been reported in other recent studies.^48, 49, 52, 53^ For example, TARID, an antisense lncRNA activates expression of the transcription factor TCF21 by inducing promoter demethylation. This appears to involve both interaction with the DNA demethylation factor GADD45A, but also possibly physical interaction of RNA with duplex DNA, forming an RNA:DNA triplex structure. Alternatively, TARID may associate with the TCF21 promoter by forming an R-loop, a characteristic structure of CpG island promoters.^49^ Our data reveal that lncRNA033862 has a positive regulator of growth factor receptor gene activity by RNA–DNA interaction, regulating SSC fate. Similar mechanisms of lncRNA-mediated control of fate determination may exist in other types of stem cells. Although multiple lncRNAs have been identified, evidence for their regulatory functions has largely relied on *in vitro* studies, and so far, an essential function of lncRNAs has not been identified *in vivo*. Mutant mice with null mutations of abundant nuclear non-coding RNAs are viable and fertile.^54, 55^ These findings suggest that lncRNAs are not essential during development or in the organism as a whole but have modulatory roles in particular tissues or under certain conditions. The *in vivo* transplantation experiments in our study strongly support the outcomes of *in vitro* analyses revealing lncRNA033862 regulates SSC fate; however, further exploration using *in vivo* genetic models is required to fully evaluate its role in the germline and other tissues.

Collectively, our study provides the first evidence for a regulatory role of lncRNAs in SSCs, demonstrating that lncRNA033862 is essential for maintenance of SSC self-renewal. Further exploring potential roles of in SSC fate regulation will be valuable to improve our understanding of the biology of SSCs, which are the only germline stem cells in the male gonad.

## Materials and Methods

### Germ cell isolation and culture

Long-term culture of SSC was established following a previously described protocol.^31^ Briefly, germ cells were isolated from 6- to 8-day-old C57BL/6 or B6;129S-Gt(Rosa) 26Sor/J mice (Jackson Laboratory, Bar Harbor, ME, USA), and Thy1-positive cells were enriched using magnetic-activated cell separation (Miltenyi Biotech, Bergisch Gladbach, Germany). Cells were plated at a density of 1.5 to 2 × 10^5^ per well on 12-well plates with mitotically inactivated STO (SIM mouse embryo-derived thioguanine- and ouabain-resistant feeder, SNLP76/7-4, ATCC, Manassas, VA, USA) feeder layers and cultured in a defined serum-free medium consisting of minimal essential medium (MEMa, Life Technology, Carlsbad, CA, USA) supplemented with 2% bovine serum albumin (BSA, Sigma-Aldrich, St. Louis, MO, USA), 20 ng/ml GDNF (R&D Systems, Minneapolis, MN, USA), 150 ng/ml GFRA1 (R&D Systems) and 1 ng/ml basic fibroblast growth factor (FGF2; BD Biosciences, San Jose, CA, USA), 10 *μ*g/ml transferrin (Sigma-Aldrich), 50 *μ*M free fatty acid mixture (5.6 mM linolenic acid, 13.4 mM oleic acid, 2.8 mM palmitoleic acid, 35.6 mM linoleic acid, 31.0 mM palmitic acid, 76.9 mM stearic acid; all from Sigma-Aldrich), 30 nM Na_2_SeO_3_ (Sigma-Aldrich), 2 mM l-glutamine (Life Technology), 50 *μ*M 2-mercaptoethanol (Sigma-Aldrich), 5 *μ*g/ml insulin (Sigma-Aldrich), 10 mM HEPES (Sigma-Aldrich) and 60 *μ*M putrescine (Sigma-Aldrich). Cultures were maintained at 37 °C in an incubator with humidified 5% CO_2_ and 95% air atmosphere. Medium was replaced every 2–3 days and cells were passaged at a ratio of 1 : 2 or 1 : 3 every 5 to 6 days.

### RNA isolation and sequencing

All reagents for RNA processing and experiments were prepared using DEPC water. For high-throughput sequencing, we used two lines of independently isolated and established SSCs that were subject to normal culture, 18 h of GDNF withdrawal and 18 h of GDNF withdrawal followed by replenishment of GDNF for 8 h. Cells were gently dislodged and collected using a pipetting method that yields germ cell preparations of high (95%) purity.^19^ Total RNA was isolated using Trizol reagent (Life Technology), followed by chloroform extraction and isopropanol precipitation. RNA preparations were depleted of rRNA and were fragmented into 200- to 500-bp molecules before synthesis of first-strand cDNA using random hexamer priming. The resulting cDNA libraries were sequenced using Illumina HiSeqTM 2000 (San Diego, CA, USA), followed by analysis and annotation of sequencing data using commercial services (BGI, Shenzhen, China). Known lncRNA species were identified by comparison with the NONCODE ncRNA library (http://noncode.org) using Cuffcompare software.^37^ To identify novel predicted lncRNAs, Coding Potential Calculator (CPC)^36^ was used.

### Data deposition

The RNA sequencing data reported in this paper have been deposited in the National Center for Biotechnology Information Gene Expression Omnibus (GEO) database (GSE66998).

### Quantitative gene expression

Primer sequences for RT-PCR and qRT-PCR are listed in Supplementary Table 2. Quantitative RT-PCR analysis was performed using the StepOne plus real-time PCR system (StepOne Plus, Life Technology) and SYBR Premix Ex Taq II mixture (Takara Bio, Otsu, Japan) in a total volume of 20 *μ*l per well. Amplification conditions were 95 °C for 30 s and 40 cycles of 95 °C for 5 s, 60 °C for 30 s and 60 °C for 1 min. Product specificity was verified by dissociation curve analysis and agarose gel electrophoresis.

### *In situ* hybridization

A 794-bp RNA hybridization probe was designed against lncRNA033862 (5′start–3′end, 1604–2397 bp) and the corresponding fragment was amplified from mouse SSC cDNA (for oligonucleotide sequences see Supplementary Table 2) and cloned into pGEM-T vector (Promega, Madison, WI, USA). *Sal*I-linearized plasmids were further purified and followed by *in vitro* transcription of Dig-labeled RNA probes according to the manufacturer instructions (Roche, Mannheim, Germany).

RNA *in situ* hybridization of 5 *μ*m cryosections of testis tissue from adult mice was performed as described previously,^56^ using 1.5 U/ml anti-digoxigenin-AP Fab fragments (Roche) and BCIP/NBT substrate (Promega) for probe detection.

### Separation of nuclear and cytoplasmic fractions

Nuclear and cytoplasmic RNA fractions were isolated from 1 × 10^6^ SSCs using the PARIS isolation kit (Life Technology) according to the manufacturer's instructions. Actin and small nuclear RNA U6 were used as controls for cytoplasmic and nuclear transcripts, respectively.

### SSC transplantation

Transplantation was performed as previously described.^41^ Briefly, 50 000 SSCs (GT-Rosa 26Sor/J) were transplanted into each testis of 129/SvCP × C57BL/6 hybrid male mice (Jackson Laboratory) that had been treated with 55 mg/kg busulfan (Sigma-Aldrich) 8 weeks before transplant to abolish endogenous spermatogenesis. Two months post transplant, testes were harvested and donor cell-derived spermatogenetic colonies identified using 5-bromo-4-chloro-3-indolyl-beta-d-galactoside (X-gal; Sigma-Aldrich) staining.

### Lentivirus-mediated lncRNA silencing

The pLKO.1 shRNA lentivirus vector and lentivirus packaging plasmids (pmd-REV and pmd-1G/pmd-LG) were provided by Dr. Chen Dahua^57^ (State Key Laboratory of Reproductive Biology, Beijing, China). The vector plasmid contains a puromycin selection site and a U6 PolIII promoter sequence for the introduction of oligonucleotides encoding shRNAs. We designed two different shRNAs for LncRNA033862 knockdown; these were mus-XLOC_033862-663shRNA directed against nucleotides 663-683 and mus-XLOC_033862-1894 shRNA directed against nucleotides 1894–1914 of lncRNA033862 (see Supplementary Table 2).

Lentivirus particles were generated by co-transfection of shRNA plasmids and lentivirus packaging plasmids into HEK293T cells using CaCl_2_ transfection following a protocol provided by Addgene (http://www.addgene.org/tools/protocols/plko/). Virus particles were harvested 48 h after transfection. For viral transduction, 300 000 SSCs were plated onto 12-well plates pre-coated with 0.1% gelatin (Sigma-Aldrich) and incubated with a 1 : 1 mixture of culture medium and viral supernatant, supplemented with 5 *μ*g/ml polybrene. After overnight transduction, cells were re-plated onto STO feeder layers and cultured in SSC medium. RNA was isolated 72 h after lentiviral transduction.

### Polysome assay

Testis lysates were prepared from 20 P10 testes in buffer containing 100 mM KCl, 0.1 % Triton X-100, 50 mM HEPES, 2 mM MgCl_2_, 10% glycerol, 1 mM DTT, 20 U/ml Protector RNase Inhibitor (Promega) and 1 × EDTA-free protease inhibitor cocktail (Roche). Lysate was kept on ice for 15 min and centrifuged at 10 000 *g* for 10 min. The supernatant was loaded on 20 to 50 % w/v linear density sucrose gradient (Gradient Master, Biocomp, Fredericton, NB,Canada) and centrifuged by 38 000 r.p.m. for 3 h (Beckman Coulter Optima L-100 XP Ultracentrifuge, Brea, CA, USA), followed by collection of RNP, 40S to 80S ribosome and polysome fractions using a piston gradient fractionator (Biocomp). For quantitative RT-PCR analysis of lncRNA and mRNA species, total RNAs were isolated from 200 *μ*l of each fraction. The efficiency of polysome separation was verified by western blot analysis of individual fractions using antibodies directed against ribosomal protein L22 (Novus, Littleton, CO, USA) and tubulin (Cell Signaling, Danvers, MA, USA).

### Chromatin isolation by RNA purification

For affinity capture of complexes containing lncRNA033862 and chromatin, we designed 10 tiled antisense probes covering the sequence of XLOC033862 using an online design tool (www.singlemoleculefish.com). Probes were numbered according to their position and separated into two pools containing either odd (1, 3, 5, 7 and 9) or even-numbered probes (2, 4, 6, 8 and 10; for sequences see Supplementary Table 2).

ChIRP was performed according to a previously published protocol.^58^ Briefly, approximately 25–30 million cells were harvested and cross-linked with 1% glutaraldehyde (Sigma-Aldrich) for 10 min prior followed by quenching with 1.25 M glycine for 5 min. Cell pellets were collected by centrifugation and lysed in a buffer containing 50 mM Tris, 10 mM EDTA, 1% SDS, 1 mM PMSF (Sigma-Aldrich), 1x Protease inhibitor (Roche) and 0.1 U/*μ*l RNAse inhibitor (Life Technology). Lysates were sonicated to shear the DNA to lengths of 100–500 bp using an Ultrasonic Broken Instrument (Branson, Shanghai, China) in a 4 °C water bath at a setting of 10 cycles for 20 s, interspersed with a rest period of 30 s. Sonicated samples were continuously centrifuged at 16 100 g for 10 min at 4 °C. Supernatants were hybridized separately with each of the two pools of 3'-biotinylated probes (GenScript, Suzhou, China; 1 *μ*l of 100 pmol/*μ*l probes per 1 ml chromatin) in a buffer consisting of 500 mM NaCl, 1%SDS, 100 mM Tris 7.0, 10 mM EDTA, 15% formamide, 1mM PMSF, 1x protease inhibitor and RNAse inhibitor at 37 °C for 4 h with gently shaking. Following hybridization, samples were incubated with Dynabeads MyOne Streptavidin C1 (Life Technology) at 37 °C for 30 min. After five washes in 2x SSC (Life Technology) supplemented with 0.5% SDS and 1mM PMSF, beads with conjugated RNP–chromatin complexes were separated using a DynaMag-2 magnetic strip (Life Technology).

### Immunofluorescence

Cultured SSCs were fixed with 4% paraformaldehyde for 15 min, and blocked with 1% BSA containing 0.1% Triton X-100 in DPBS buffer for 1 h at room temperature. After incubation with primary antibodies against PLZF (1:100; R&D Systems) and LIN28A (1 : 100; Abcam, Cambridge, MA, USA) for 2–3 h at room temperature, cells were washed 3x with DPBS, followed by incubation with secondary antibodies (bovine anti-goat-TRITC 1 : 1000 and donkey anti-rabbit-FITC 1 : 1000) for 1–2 h. Samples were analyzed using confocal microscopy (LSM 700, Zeiss, Pleasanton, CA, USA).

### Data processes and statistical analysis

Image data were assembled using Adobe Photoshop 7.0 software (Adobe, San Jose, CA, USA). LncRNA expression analyses were performed by BIG Company (BGI, Shenzhen, China) as reported previously;^59^ *P*-values correspond to differential gene expression tests and false discovery rate (FDR) was used to determine the threshold of *P*-value in multiple tests. Sequencing, gene expression, transfection and transplantation experiments were performed in duplicate or triplicate using independently established SSCs cultures. Differences between groups were determined by one-way ANOVA using SPSS 16.0 statistical software (SPSS, Armonk, NY, USA). A difference was considered significant when the *P*-value was <0.05.

## Abbreviations

SPCs: spermatogonial progenitor cells
SSCs: spermatogonial stem cells
lncRNA: long non-coding RNA
GDNF: glial cell-derived neurotrophic factor
Gfra1: GDNF receptor alpha1
CHIRP: chromatin isolation by RNA purification
shRNA: small hairpin RNA

## Supplementary Material

Supplementary Informations
