# Supplementary material for: A long non-coding RNA interacts with Gfra1 and maintains survival of mouse spermatogonial stem cells
Source: Cell Death Dis. 2016 Mar 10;7(3):e2140–. doi: 10.1038/cddis.2016.24 (PMC4823932; doi:10.1038/cddis.2016.24)
Supplement: Supplementary Informations [file cddis201624x1.pdf]

# A long non-coding RNA interacts with Gfra1 and maintains survival of mouse spermatogonial stem cells

Lufan Li[1*], Min Wang[1*], Mei Wang[1*], Xiaoxi Wu[1*], Lei Geng[1], Yuanyuan Xue[1], Xiang Wei[1], Yuanyuan Jia,[1] Xin Wu[1]

Summary of supplementary data:

This supplementary information includes 9 figures and 2 tables supporting the main text, detailed data from each supplementary figure and table has been mentioned in the main text.

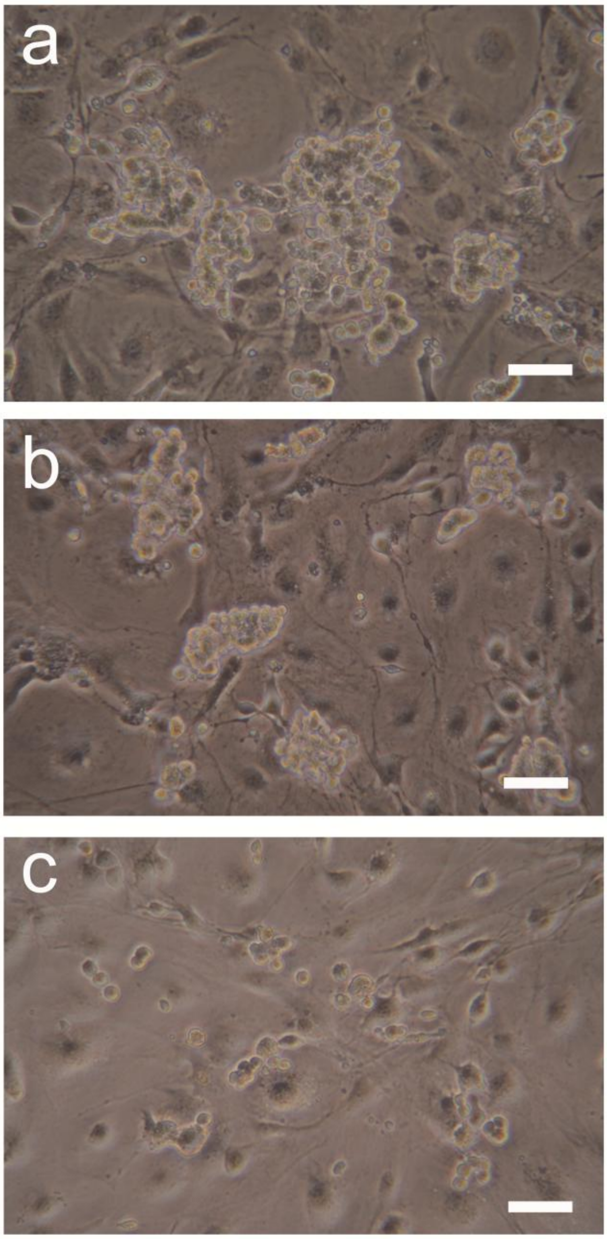


**Supplementary Figure 1. Clump-forming SSC cultures.** (**a**) In the presence of GDNF, SSC clumps exhibited typical grape-shaped appearance. (**b**) FGF2 withdrawal from the culture medium resulted in slower proliferation but a net increase in cell numbers (see Fig. 1d); upon FGF2 withdrawal, colonies were smaller but retained clump-forming morphology. (**c**) In contrast, GDNF withdrawal resulted in disappearance of clump forming cells within 7 days. Scale bar=100µm.

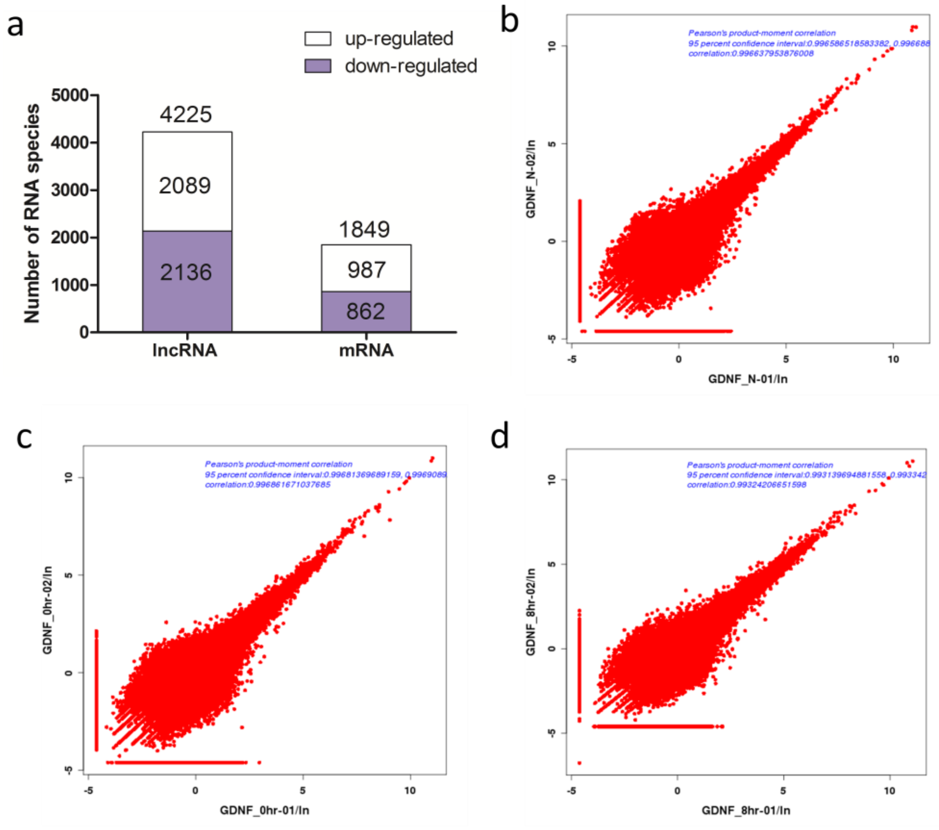


**Supplementary Figure 2. RNA transcripts regulated by GDNF refreshment and Pearson correlation between samples.** (**a**) Comparable proportions of mRNA and lncRNAs (2089/2136 vs 987/862) were up- and down-regulated upon GDNF refreshment. (**b**) Pearson correlation value was calculated between two normal cultured samples (Normal, 0.996), two 18h GDNF withdrawal samples (0h, 0.996) and two 8h GDNF refreshment samples(8h, 0.993)

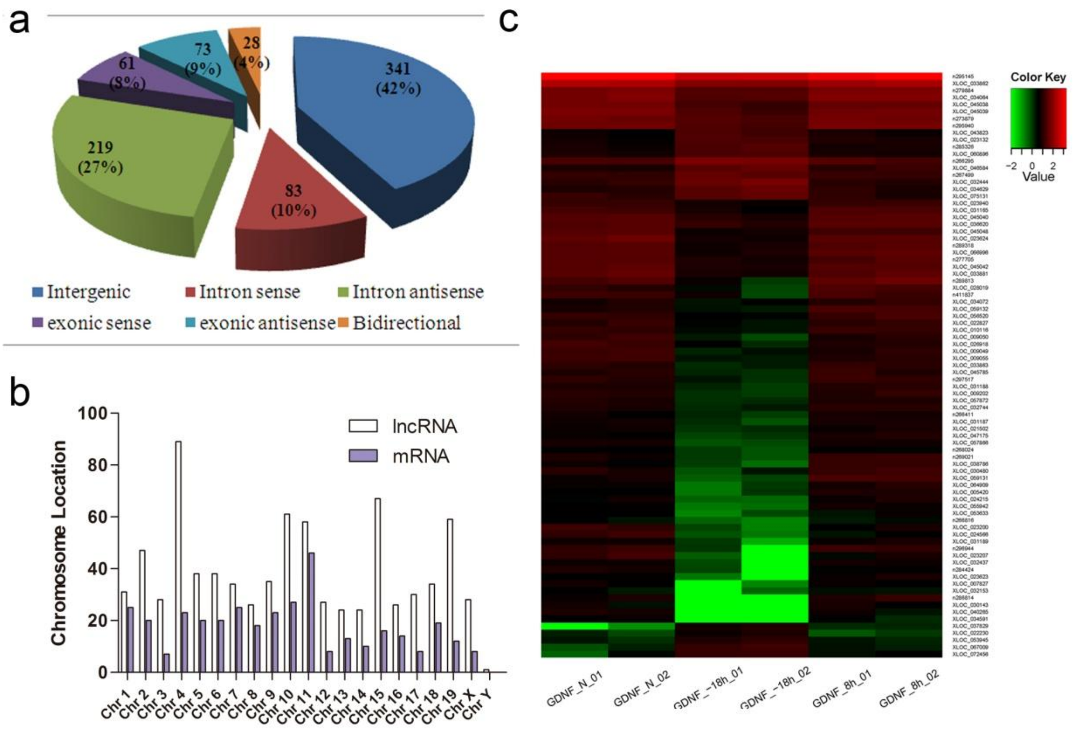


**Supplementary Figure 3. Characterization of GDNF-regulated lncRNAs.** (**a**) Genomic context of lncRNAs regulated by GDNF. Most of the 805 lncRNAs were intergenic, intron antisense, or intron sense. (**b**) The 362 mRNAs and 805 lncRNA transcripts found to be regulated by GDNF were randomly derived from all mouse chromosomes. (**c**) Heat map of 83 lncRNAs with FDR<0.05 and at least 2-fold change in expression level in individual samples of GDNF treated SSCs.

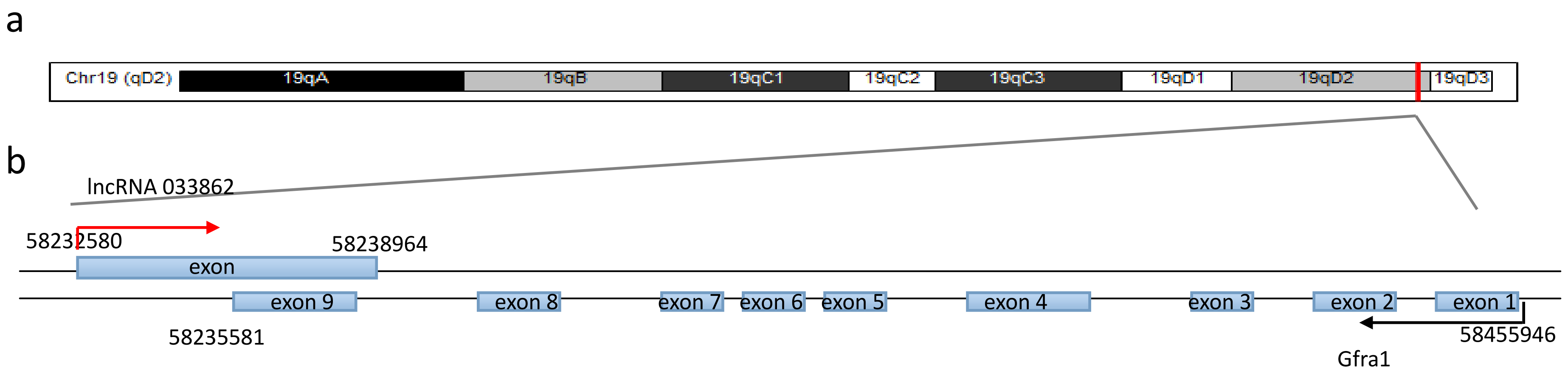


c

5’-GGGGAGGGAGAGGGAGAGGGAGAGGGAGAGGAGGTTGCCTTTACCTGTCCTTGCAACAATCACCATACTGATCTAGTTTCTGGTGACTGTTTTTTACTATTGTAAAATAAATA

AATAAATATAAATAAGACAAACCCAAACCATCTGAGCTTCCTATAGTAGGGCATCAATTTAGGCTTCCTACTCTTCGTCCCAATTCACTTGAACTTTGGGGCCCGTGTTATAC

GTGGTGGATATTTGAGTTCCTTGTACAACTTCAGTTTTGCTATGTATATAGGCCCTGATTAAGATGGCCTGTGAGATCGGGAGGGACAGCAGAGGGAAAGCGGTCTGAAAAAA

TGAATGAACGTGATTGTGTGCGATGATGTAATCACGAGACCCATGAGCTATGATGTAATCACGAGACCTATTAGCTGTGATGACATAATCACGAGGCCCATTATTTTGTATGC

TAAAACCATTTTAAAAGGAGCCCTACATTTGCTGTCCTTAGCCAAAGCAGCTCTAGAGTCCCTTCTTCCATAGCACCTAAGCTGCCTCAGATCACTGTGACCTCCCCACTGGC

ACACCTGTACAGCCAAGCTCATGACCATTTGAGGCTCAAACTAAGGGTGGCTTTTCTGTGTCCAGATAGGGTCTGTGATATGTCTCTTGGAGACCATGTCCATAGTCTCTGTA

ACTATACAGAGAACCTCTGG CAGGTATCAGAGACAT

```
TTATAAAGAGGGCATCAAACTGAAGGATTTCAACAATCAGCAATAGTTAATGAAGATTAAAATCTCTGTAGGCTCACTGTTCTGGTAGATATCAATCTATTGGGATTAGCTTG
|||||
AATAT

GGACATGGGGATGGGTGTTTATGTCTCAGTTCTCAGCAGGAGACATGGGTATATGAACACCTTCCTCGGCCCAGGGCACTCTCAGTATTGTAATAGTCTCCTGAAAATGTCAT
                                                                         ||||||||||||||||||||
                                                                         CCCGTGAGAGTCATAACATT

TCAAGGCCAGCCCAGCTATGTGGCTTGACAGCTCTTGCTGCCATGGGGTAGAATGAAGCCCTTTGGTGTGATTTTCAGGCGACTGGCATTTTAAAAGTACCTACTATGGGTTA

TACCTACAAAGGAAGGGAATGAGCTGGGAGCAGTGTTCAAAGTGAAACTGTCTCTGGCCAGGCTCCTGGCATCTTCAATAAAATGAAAGTGCGGATGGGTAAGAAAGTTCCAG
                                                                                                 ||||||||||||||||
                                                                                                 CCATTCTTTCAAGGTC

ACTGTGAAGCAAGAAAGCCCAGAGAATCCCTTCTATTGATGCTGGTGCTGAACTTGGCAGCTGCCCTTGAGTAAAAATTACCTCCTCCTCATGGCAGTGGGACAAGATGCTGA
||||
TGAC

TACCGGCACCTGCCAGAGGCAGACAGCCCAAGTCACAGCTGCGTTGGTCAAATGAGCTTGGAGACAGACCCCAAGGGCTTCTGGGGGCCTTGATGACTATCGGCAGGCTGCTG

CACTCTGCAGGCAGTTTATTGGAACTGGGTTCTTATGCCTTCCCCACCCCTCTGGGCTGGGTCTCAGGACTCATCAGAAATGCAGGTGGCATATCTAGATGATCAGCCATGCT

GAGCTGCCTCTTGGAAAAAGTATGGATTATCAGAGAGCTCCAGAGATATCAAAGGGATCCCCATGAGGGTATAGGAGGCAAGGCACTAAAGTGAAAAAGAGGGAATCTCAAGA
```

```
GAAGGTAGGGGAATGCGTATCTGACTTGAGGCTTTCATCATCTGGCCTCTGAGACACCTCTTCTTCCACAAAATGAGCATCCGATAGAAGGAGGATGGATTTTGGCAGCATTG
                                                                ||||||||||||||||||||
                                                                AAGGTGTTTTACTCGTAGGC

AGCTCAGATCGATGGGTGAAGAATAGATGGGAGGGGGGTTCCATGATATGCGTTGGCAATAACAGAAAAGGGAGGCATGGAGGCTTTGGTAAGCAACGGGAAGATAACAGAAC

CAATCGCATCTTAAGACATCTCTCCTTAGGAAATCAACATCCTGCAGGGTACATGCCAAGCCACAGGCAGCTGCAACCCCATGGGAGTAAGGCCTCTGCTATTATTCCAAGGT
                                                                                      ||||||||||||||||||||
                                                                                      TCATTCCGGAGACGATAATAA

ACAGTTCTCCATGGAGAATCCTAGACAACAGACTTAAGAGACATGAGAGTCCTTGCTCGGGACAAGAGAGAAGAAATGTAGCTTCCAGGGTTGTTGAAGAAATATCCCATCCC

TGGCTTTTTACTTTAGTTTCTAAAAGCAAAGCCCTCCATTAGTCACTCAAACATGAGTTCTAGCGCCCTCTCTACAGAGGACCTCCTGCTACTTCAGGACACAGGCTATTTTT
                            ||||||||||||||||||||
                            TTTCGGGAGGTAATCAGTGA

TGTTTTTAGAAGAATGTCTTTATCAATAAGAAACATCAGCAGAGCAAGAGATCTACTGTGACTCTTTATTGCGCCTTAATTTTGCCTCGTTTCTCCAAGCCCTGTGAGCTCAG

GGCAGCTGGGCCTCCTGGTGTCATGGTGCCCAGACTATGACAATTCATTTCCATCTGTTGCCACTGTCTATGGTCTTTCCTAAAAAGATGGATATCTCAAGGTCTTATTTGCT

TAAAGAGCACAGCCAGGACAACAGAGAGTAGGGGCATGGATCTCGGAGACCAAGAGCAGCATCCTAAATGCAGAGCATGAGCTGATTTAAGCAACCCGCTCTGCTCTGCACTT

TGACTCAAATAAGCCGTGTCTGACAATATGAACAAAAGCTTATTATGTTCCAACCGATGGGTCCTCTGAGTCCACCATTTTGTAAGTAATAACTGAAGTGCATTTATGTTGGA

ATCTAAGGAAATAATTTGCTAATTACATATCGCCCTCACTGCTTTTACGTCTCTTTTTCTATAGTCACTGATTCAAACAGGCTACCACCCTTTCTCATTTCTCCAACTCAGCA
```

GATGGGGTGTTTGGGGGGTGTCTGATTTAGAGACCAGAAGGCTCATGGGAAATGTGGTGCAGCTAAGAATGACCCAGCCTTGACACACACACACACACACACACACACACACA

CACACACACACACACACACACACTAAACTCGGCTCTGGAGGGTCCTCGTGCTGTGTTGGGATGAACTTGAAGCTGTAACTACTAAGACATTTCCATTTAGCTACAAGATATGGAA

| | | | | | | | | | | | | | | | | | | |

GACACAACCCTACTTGAACT

CTGCCTTAAAAATAACATGGAAACCACCCTCAAATTACATCTAGACACAATTCGCGTTACTTGTTGAAAAATAACCTATGCCATCTTTAATGTCTATATTTTTTTTATTTGAG

GTTTGGAAAAAAATCAGATGTGGAAATATAAACAGCCAGCATTCCAAATTCCCTCTGTGTTGTGCACTTGTCCTCTCGTGTGCTCTCTTAAAAAGACTCTCAAACTCCATCTT

| | | | | | | | | | | | | | | | | | | |

TGTCGGTCGTAAGGTTTAAG

CTCAAGGACATTCAAGGTCAAACAGAATGAATGTGCTTCTGCTCAAAGTGGCTCCATCACCTTTGTCTCTCCCCGACGTAGGCTGGATTCCGAGTCACTGTGTGGACGCAACT

GTCAGTAAAATCAACTTCGCAAAGGACTTTCTGTAAGGAACCCAACCTCAGCAGACGAAAACTGTCTCTGACCAGGGAAAGCAGGGAATCACTTCCGCTCATGGGCGGCGCTC

CAAGAGGAAGTCGAGTGCATAACAGATGGCCAAGTTTCCCTGTTCTAGTCTTGAGATGAAGCAAGCGCTTTGTCGAGGAGGCCACAGCAAATGGAAGACCTATATGCTTGCCT

TGGTTTTGTAGCCATCTCTGACATGTCCCTAAATGGGGTCCTTCTCACTATTTTATTTCATCAGCAACTTGTTTTCAAATCGAGGATTTGGTAACTCTCAGTCGCTTTTATCT

CTAAAGGAAAATGCAATTTTTACTGGTGACAGTGCTCTTCAAAGTTTAATTTGTATGTTGCCTATCTACGAGTTCTTGCTGAAGTCCCCTTGTTAGCACAGGGCTCCTGTTTC

CTCTCTGACCCTCTGAAGCTAATGACAGCTGCATTATACTGCAGAGGTAATCAATAGCCTCCTATGGCGGATCACATCCAATCAGACCCTTATGAAATTAGTGCAGTGGAGAT

ATATGTATATATCTTAATTAAAACGAAAATATGATCAGTAAGAGATTATGTTCCCATTCATGACCGCCATTCTGTTCCGACACATCGAATATGTTTATTCAATGTATGAGTCT

TCATGGAACGTCCCACTGATCAGGTACACTTGGATGTTGGATGCATTCCCCTCAAAAACATTGTGAAGGGGAAAAAGAGGTTGCTACTGAGTATTTTACATTGTTATTCAAAA

ACATTTCAATATAAATGCACTTCAGACACCAGAGGAGTCCCTCCCAGGTGAATCGTTATTTATTATCCCAAAGGCAAACCTCAGAGAGCAGAGCGGGTATCAGGAGACCGTGC

ACCAGGGCTCCCTGCTCTGTGGGAGGGTGAGGGGCCAGATCATACTTCTAGGGCTCAAGGGAAGGAAGATTCTCCCCCCCCCCCCTTATTCACTTTGCAGGAAACATGGTTGC

CAGTACGCAACCATTAACAAATTCTGAGCCTGACCATGTAAAGCAGTCTTTAGATATATGCCTAAGGGTCTGCCCAGGCAAGGAGGAGGGCTCCAGGAGATGGTGTGTGTGTC

| | | | | | | | | | | | | | | | | | | |

TAAGACTCGGACTGGTACAT

TTTTCTCGAGCGTCCCCTCCTGCTCTGTGTACTTGTGCCATTCAGTTCACCTTCCCAGACCCTGTGCTGGCCCTCTAGATCCATAACCTACCATTCCTTTTGTGTGTGACAGT

CTGTCATGAAGGACACAGAGCAAAATACAGGAGATACTTGGTGACCTTGAATCCAAACTGTTCTAAGTTGTGGTCAAAAGCAAAGAAACTTCCATGTGCCCTGGCAGGACTCA

ACTGTGAAAGAGAGGGCAGCATATCTCCATCCCAGCCAGTCCTAATCCAAGCTGTCTCTGCCAGACAACATGAAAACCCATGTACTGGGGCTATATCAACTGCCAGAAGCGCT

GTCTACTGTGGCACCCTTTACAAGTATCTAACCATTCCTTACATATAGGCAGCCTTGATCTCAAGACAGATGAGACATGGGCTGGCTGCTCTATTTCATCCTGCCTGTCCACA

| | | | | | | | | | | | | | | | | | | |

GTATATCCGTCGGAACTAGA

GAAATGTGATGTGGCTTTCTGCTAAAGCACTGGGCTTCTTGGGGGCCTGCTTTTAATGAGAGCCATATGCATGAAGTTGATGCTGGAGCTAGTATTACTGATGAGGGGCAAGG

GCTGTGTGTATTAAACCATCATTAAAACATGGGACCGGTATCGCTGCCTTCAAAATGTAGAAAGCTAACAGTTGGAGAGCTGACAGTTGGTAATGCTTGTTCTACATGTGACC

TCGTAAGTGTAAGAAGTGGTAAGAGTTGATCTTCAGCTACATTCAGCTTCTCACTCAAGCAAAGCTCAGGAGAGCGCACCAGTGAACAATCCCGGGTGCTGTGCAAGGACCAC

ACCCACTCTCCTCTACTGGGCTAAATGGTAGCTTCTACATCTGGATGTGACCAGGGACTTTTTTGTTTTGTTGGGGATCACATAAACACTCTGTGGAGAGCATACATAGAACG

GTGAACGTTAGTACAAGATAGAGTCTGCAAAGTCCACTCTTGAGGCCAACCCCCAATTGTCATCCATCCACACTAGGCTGCAGTCATGTCTTTAGAATCCAACGCTATTAAAT

AAGATCCGGAAGAATCTCTATCAATAGCATCTCCTCCTCCTAGCCAGTAGAGAGAACAGGGTTCAAGCTGTTAAAACTATCATCTTCATCATCACCACCACCATCACCATCAA

AAACATAAGCTCAGCTTGCTTTACAGTCCAGGTTACAAACAGTCCCCTGAAGCCACACCATGCCCCAGAGTTTTCTGAGTTTGGAATTAGCCCTGTAGCAGTTCTTCAACAGA

AGCCTGTGAGGGCGGCAAAAAGCTTTCTTAAAAACAACAAAGGAGTTCCCGCTGGGTGAAACTGCTTCTCAACCGAGCTTTTAAAACTGGATTTTCAGATATACAAGAGGACA

GGGGACAGAATACTTGGTTTTCTTTTGTCTTTTCATACTGGATGCAGCTACGATGTTTCTGCCAATGATACAGACAACAGGGCAGCCAGAGCGGTGAACACCATCACCGGCAG

TGAGCTCAGACCGCAGCTGGGAGGAGCAGCCATTGATTTTGTGGTTATGTGGCTGGAGGCACCAGCGAGACCATCCTTTCCGTAATCATTCTGTAAGAAACAATGTTTTCATT

ATCACCCACAGCACATGCCCACTTCCTCAGGTTGGATTTTACCCGTGTTCTCTAACCCTACTTCCCTGATAGCTCCTTCCCAACAAGCTGGCCTGAGTGTTAACCACTGAGAC

TGAACTGGCTAACTTTTAGTTCTGAGATGGGACCATGAACGTTCGTAAGATGTGGATGGATCCTTTGGATCAACTGCTAGCCAATGTTTGCTGTTTGATCTGTGATACATTCT

||||||||||||||||||||

ACTTGCAAGCATTCTACACC

TTCTGATGGGAAATGTTTAAACCCAGGGGTCACTGCCTTTATGTCTTTTTCTAGACCACCTAATAGCTATTACCTCCTGGCTTCCTTCTGACTCTCCCCCCACTGGGCAAGGA

AAGCAGGAAGACGAGTCTGGGGAAGTGTAGCCCATGGAGACACAGGTGGATCAAAA-3’

**Supplementary Figure 4. Structure of lncRNA033862. (a)** LncRNA033862 is a 6384bp RNA transcript transcribed from mouse chromosome 19 with a single exon (red region). **(b)** LncRNA033862 (red arrow) is a partial antisense transcript of *Gfra1* (transcriptional start marked by black arrow) that initiates 3 kb downstream of *Gfra1* exon 9 and includes exon 9 (2935 bp) and the intronic region between exons 8 and 9 (449 bp). **(c)** Full sequence of lncRNA033862 illustrating the sequence representing a partial antisense transcript to *Gfra1* exon 9 (yellow highlighting), the amplicon used for detection by RT-PCR detection (red), oligos for shRNA inhibition (blue), probes for chromatin isolation by RNA purification (CHIRP, green), and the probe for in situ RNA hybridization (purple; with sense sequence representing the lncRNA033862-specific probe, and antisense sequence used as probe control).

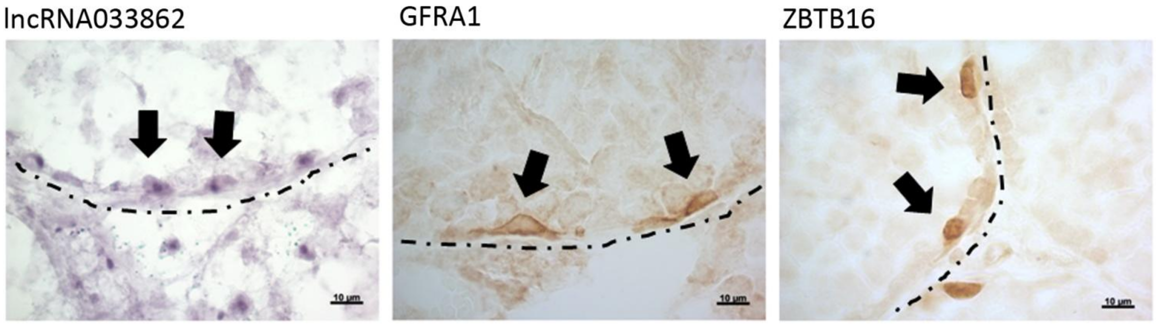


**Supplementary Figure 5. LncRNA033862 localizes to spermatogonia.** RNA in situ hybridisation of lncRNA033862 in testis cryosections from 4 to 5-month-old adult mice was performed with a 794 bp RNA probe. The complementary sequence to the probe was used as sense control (see figure 3e). Strong staining (NTB/BCIP substrate, brownish red without ethanol rinse) was found in the nuclear region of spermatogonia-like cells on the basal membrane of seminiferous tubules. These cells express the spermatogonia marker GFRA1 and undifferentiated germ cell marker ZBTB16 (PLZF), visualized by immunostaining (DAB substrate, yellowish-brown), and thus represent type A spermatogonia. Size bar, 10 $\mu$M.

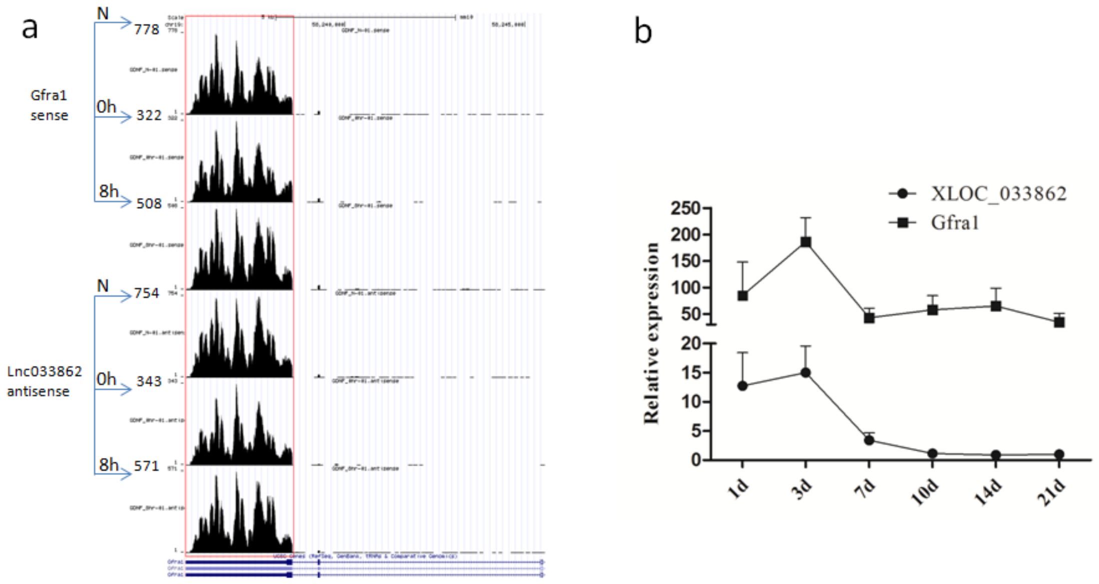


**Supplementary Figure 6. Transcript levels of Gfra1 and lncRNA033862 correlate in cell lines in response to GDNF and in vivo.** (**a**) Correlation of *Gfra1* and lncRNA033862 transcripts in RNA-seq experiments visualized by a representative snapshot (from cell line#1; generated by http://genome.ucsc.edu) of the exon 9 region of *Gfra1*. (**b**) Quantitative RT-PCR reveals that lncRNA033862 transcript and *Gfra1* mRNA correlate significantly in the testes from postnatal day 1 to day 21 (Pearson correlation: 0.538, p<0.05). Error bars indicate STDEV.

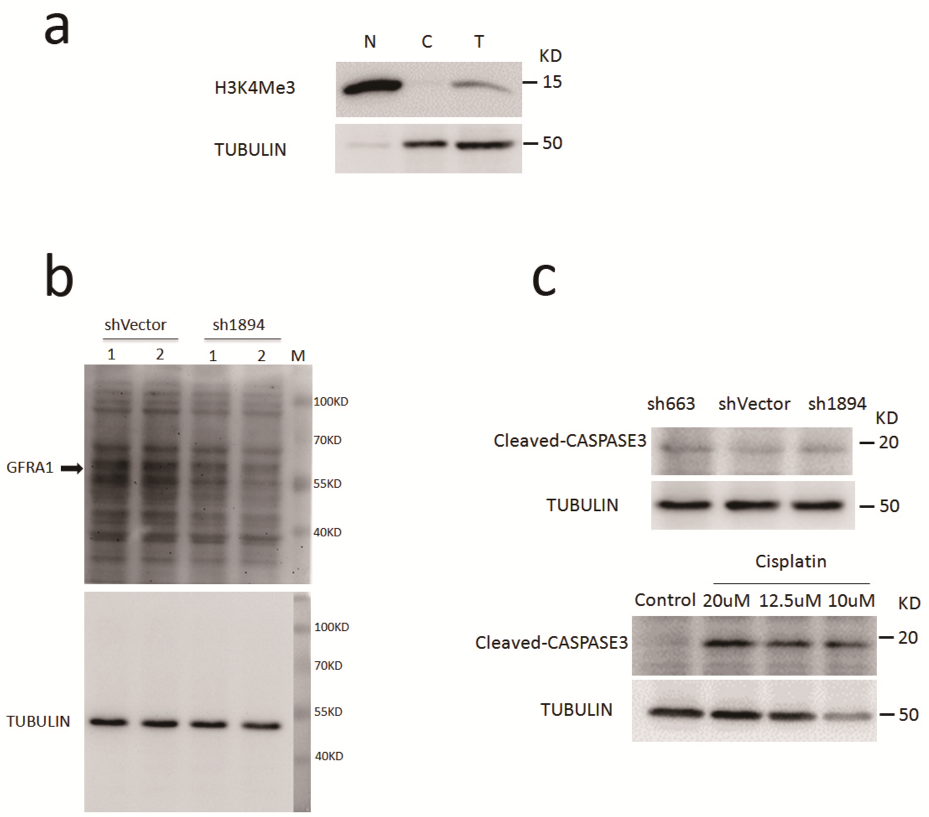


**Supplementary Figure 7. Protein level identification** (**a**) Western blot to verify the cell fractionation, compared to whole cell lysis, β-TUBULIN (Cell Signaling, Cat#:2128S, 55kDa) is enriched in cytoplasma fraction and H3K4Me3 (ABclonal, Cat#: A2357, 15kDa) is enriched in nuclear fraction. (**b**) Western blot to detect Protein levels of GFRA1 following lncRNA033862 knockdown. Tubulin served as control. (**c**) Western blot to detect the cleaved-CASPASE3 as an apoptotic marker. Cultured germ cells have higher intensity of cleaved-CASPASE3 (Cell signaling Cat No:9664 17,19 kDa) following lncRNA033862 knockdown. Cisplatin-induced apoptosis served as control.

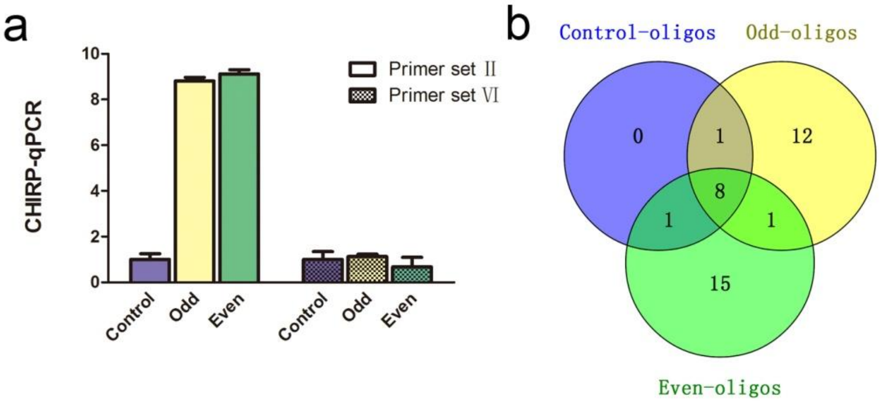


c

| Control-oligos | Odd set-oligos | | Even set-oligos | |
|---|---|---|---|---|
| 22100 a10C04Rik | 2210010C04Rik | Scamp1 | 2210010C04Rik | Scamp1 |
| Ubc | Ubc | Lyz1 | Ubc | Hspb8 |
| Amy1 | Amy1 | Ldha | Amy1 | Pm20d1 |
| Hist1h4a | Hist1h4a | Foxn2 | Hist1h4a | Gxylt1 |
| Tubb4b | Mup9 | Tuba3a | Tubb4b | Pvalb |
| Mup9 | Actb | Dnajb14 | Mup9 | Obp1b |
| Actb | Ybx2 | Dmrta1 | Actb | Brca1 |
| Ybx2 | Fam107a | Unc13a | Ybx2 | Rec114 |
| Cacna1d | Cacna1d | Birc3 | Cacna1d | Pnliprp2 |
| | Camk1 | Ghitm | Akt2 | Ltf |
| | Blmh | Enam | Gm28045 | Jup |
| | | | 6430550D23Rik | Cdan1 |
| | | | | Dsp |

**Supplementary Figure 8. LncRNA033862 associated proteins.** (**a**) ChIRP-qPCR with primer set II (amplifying the regions of *Gfra1* that overlap with lncRNA033862) resulted in an up to 8.8 fold enrichment of DNA fragments captured using the odd set of tiled oligos, and an 9.1 fold enrichment of fragments captured using the even set of oligos; in contrast, amplification with primer set VI (amplifying the regions of *Gfra1* that do not overlap with lncRNA033862), did not result in enrichment of DNA fragments. Non-bead-conjugated oligos served as a negative pull-down control. (**b**) Eluted protein was subject to protein mass spectometry (Modle, Ultraflex II MALDI-TOF/TOF, Bruker Daltonics Co.). Venn diagram analysis demonstrated that only one protein, SCAMP1, was common to complexes pulled down using odd (yellow) and even oligonucleotide pools (green). (**c**) List of protein candidates identified in (**b**). Error bars indicate STDEV.

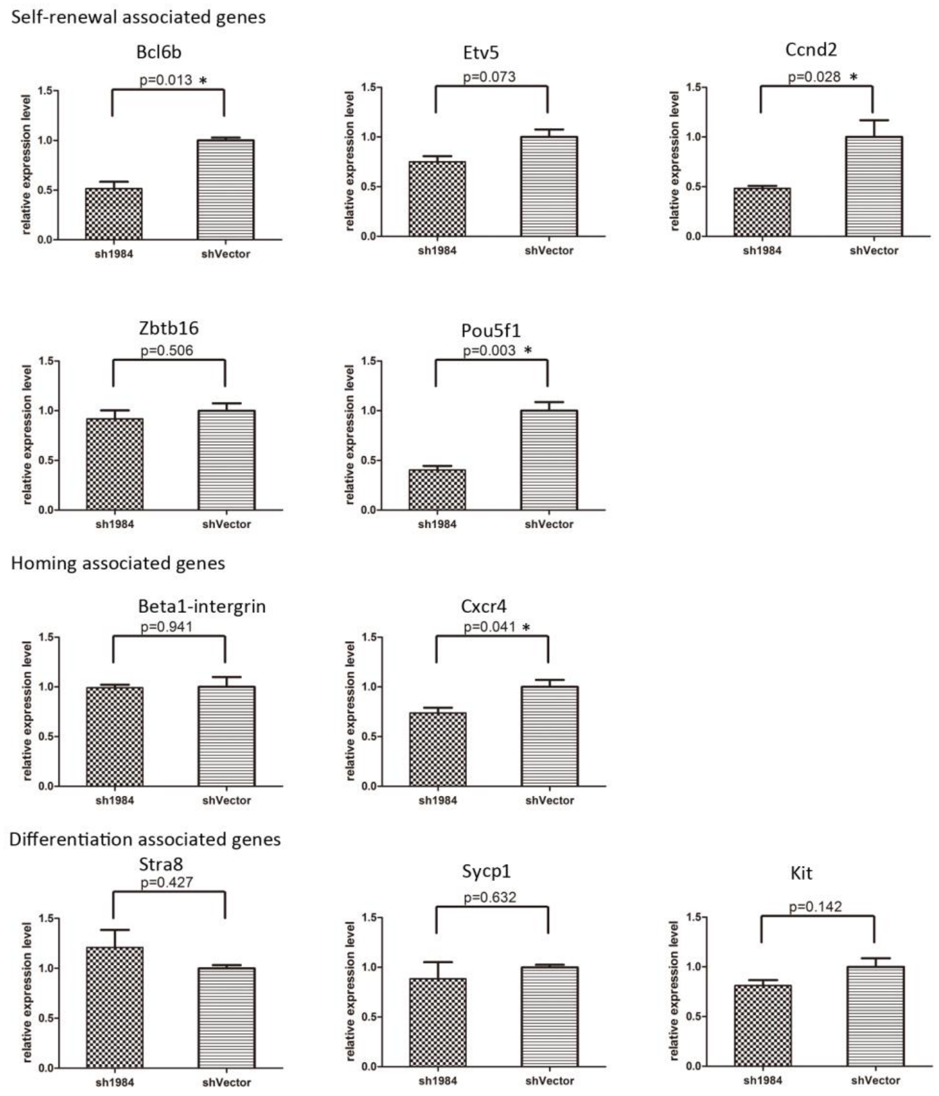


**Supplementary Figure 9. LncRNA033862 knockdown results in downregulation of self-renewal associated genes in SSC cultures.** Quantitative RT-PCR for transcripts of 10 SSC-associated genes in cultured SSCs after lncRNA033862 knockdown; shVector served as control. Asterisks mark significant differences (t-test, $p<0.05$). Transcript levels of the self-renewal genes *Bcl6b*, *Ccnd2* and *Pou5f1*(*Oct4*) were significantly decreased, whereas *Zbtb16* was not affected. Among genes associated with homing, *Beta1 intergrin* mRNA levels were not affected by lncRNA033862 knockdown whereas *Cxcr4* mRNA was decreased. Transcript levels of representative genes reflecting SSC differentiation (*Stra8*, *Sypc1*, *Kit*)

were found at extremely low level and not significantly changed after knockdown of lncRNA033862. Error bars indicate STDEV.

**Supplemental Table 1 Top one hundred lncRNA species identified in SSCs**

| Number | ID* | Length (bp) | baseMean_N | baseMean _0h | baseMean _8h | P value (N vs 0h) | FDR (N vs 0h) | P value (0h vs 8h) | FDR (0h vs 8h) |
|---|---|---|---|---|---|---|---|---|---|
| 1 | n422895 | 1612 | 1273548.16 | 1437399.59 | 1214145.10 | 0.42873333 | 1 | 0.273346 | 1 |
| 2 | n294267 | 838 | 748357.74 | 833004.00 | 676430.90 | 0.48790179 | 1 | 0.169625 | 1 |
| 3 | n422898 | 2883 | 741083.51 | 761130.33 | 657142.60 | 0.866812732 | 1 | 0.336383 | 1 |
| 4 | n422896 | 690 | 644416.83 | 669233.96 | 648034.31 | 0.799877019 | 1 | 0.840097 | 1 |
| 5 | n422893 | 2555 | 523569.47 | 514334.60 | 412638.36 | 0.898396814 | 1 | 0.149433 | 0.992435 |
| 6 | n422894 | 1085 | 333257.76 | 322786.72 | 258364.84 | 0.833361282 | 1 | 0.142017 | 0.978771 |
| 7 | n423941 | 2351 | 171046.11 | 183395.84 | 140084.10 | 0.662327748 | 1 | 0.080228 | 0.822087 |
| 8 | n272737 | 379 | 129115.85 | 138330.51 | 132485.58 | 0.638474582 | 1 | 0.773793 | 1 |
| 9 | n272837 | 686 | 116293.90 | 107380.49 | 100710.67 | 0.660055275 | 1 | 0.691568 | 1 |
| 10 | n265149 | 1424 | 21471.55 | 22734.24 | 27298.14 | 0.820730364 | 1 | 0.429801 | 1 |
| 11 | n297626 | 939 | 10044.88 | 10669.85 | 9352.97 | 0.70300895 | 1 | 0.404661 | 1 |
| 12 | n264684 | 1074 | 9203.65 | 10222.70 | 9421.97 | 0.47918537 | 1 | 0.570529 | 1 |
| 13 | XLOC_033862 | 6384 | 7136.16 | 2504.45 | 5249.89 | 3.07E-11 | 1.41E-07 | 2.02E-06 | 0.000963 |
| 14 | XLOC_011180 | 8956 | 6768.62 | 6779.67 | 7535.08 | 0.993208372 | 1 | 0.509019 | 1 |
| 15 | n269743 | 889 | 6691.27 | 8497.36 | 8797.49 | 0.189756875 | 1 | 0.819619 | 1 |
| 16 | n267238 | 2574 | 6604.58 | 7881.19 | 6183.96 | 0.247681578 | 1 | 0.11696 | 0.930178 |
| 17 | n416847 | 2478 | 6357.99 | 6312.47 | 6289.03 | 0.970035581 | 1 | 0.971025 | 1 |
| 18 | n413703 | 6621 | 6095.66 | 5638.19 | 6170.83 | 0.676767341 | 1 | 0.599846 | 1 |
| 19 | XLOC_003490 | 4676 | 6042.42 | 5001.15 | 4265.19 | 0.574955552 | 1 | 0.605794 | 1 |
| 20 | n295145 | 811 | 5713.16 | 1902.44 | 4872.34 | 2.95E-07 | 0.000311 | 1.07E-05 | 0.003332 |
| 21 | n344450 | 2219 | 5418.73 | 2953.01 | 2215.51 | 0.000110496 | 0.040788 | 0.06304 | 0.757831 |
| 22 | XLOC_069106 | 1590 | 5417.97 | 5147.09 | 3891.05 | 0.889220141 | 1 | 0.499353 | 1 |
| 23 | n421342 | 2539 | 5379.19 | 5155.25 | 4377.05 | 0.79486504 | 1 | 0.282505 | 1 |

| | | | | | | | | | |
|---|---|---|---|---|---|---|---|---|---|
| 24 | n273021 | 1563 | 5323.17 | 6292.76 | 5634.85 | 0.478214873 | 1 | 0.659908 | 1 |
| 25 | n413706 | 2026 | 5304.86 | 5739.79 | 7305.06 | 0.58373003 | 1 | 0.113023 | 0.921513 |
| 26 | n284720 | 1916 | 5107.58 | 5322.01 | 3983.14 | 0.813547565 | 1 | 0.062031 | 0.754274 |
| 27 | XLOC_074383 | 3288 | 4883.28 | 5708.51 | 4272.40 | 0.312959647 | 1 | 0.060905 | 0.749052 |
| 28 | XLOC_015015 | 3457 | 4823.67 | 3328.93 | 4831.98 | 0.016698068 | 0.767832 | 0.016856 | 0.428677 |
| 29 | n289939 | 2655 | 4763.88 | 6089.74 | 5622.93 | 0.21676011 | 1 | 0.681857 | 1 |
| 30 | n265035 | 4068 | 4650.39 | 4776.68 | 3899.97 | 0.88009913 | 1 | 0.200625 | 1 |
| 31 | n424047 | 1787 | 4184.45 | 4549.31 | 6360.57 | 0.634159873 | 1 | 0.031283 | 0.572833 |
| 32 | n283494 | 3371 | 4071.44 | 3497.67 | 4075.25 | 0.336595586 | 1 | 0.327732 | 1 |
| 33 | n295169 | 2017 | 3941.74 | 3756.83 | 4235.29 | 0.754383144 | 1 | 0.421289 | 1 |
| 34 | XLOC_046462 | 3148 | 3902.73 | 5061.07 | 3815.21 | 0.093192102 | 1 | 0.067979 | 0.778099 |
| 35 | n266044 | 2025 | 3829.98 | 3840.21 | 6418.87 | 0.938367715 | 1 | 0.001003 | 0.085527 |
| 36 | n343786 | 5845 | 3663.60 | 3128.00 | 3772.35 | 0.313639637 | 1 | 0.226665 | 1 |
| 37 | n286390 | 4961 | 3580.73 | 2583.19 | 1867.13 | 0.077037826 | 1 | 0.073628 | 0.797344 |
| 38 | n271003 | 4229 | 3531.59 | 3214.97 | 3727.91 | 0.58667201 | 1 | 0.357894 | 1 |
| 39 | XLOC_033631 | 9707 | 3493.21 | 3189.57 | 3334.63 | 0.554764071 | 1 | 0.759315 | 1 |
| 40 | n293679 | 3209 | 3483.66 | 3337.82 | 2738.38 | 0.865711934 | 1 | 0.427239 | 1 |
| 41 | n273670 | 3730 | 3480.01 | 3071.75 | 3411.29 | 0.425381936 | 1 | 0.509654 | 1 |
| 42 | n293512 | 2815 | 3474.52 | 2977.41 | 3016.18 | 0.363355755 | 1 | 0.909591 | 1 |
| 43 | n343833 | 721 | 3353.34 | 3631.58 | 3730.01 | 0.709415119 | 1 | 0.939475 | 1 |
| 44 | XLOC_051561 | 3974 | 3327.20 | 2160.26 | 3276.66 | 0.005567648 | 0.461815 | 0.007419 | 0.275908 |
| 45 | n265642 | 4871 | 3230.14 | 2304.17 | 1871.05 | 0.032698136 | 0.993918 | 0.185585 | 1 |
| 46 | XLOC_060814 | 5118 | 3179.59 | 2899.56 | 2540.08 | 0.553875881 | 1 | 0.405996 | 1 |
| 47 | n264998 | 3889 | 3139.12 | 2346.62 | 2804.41 | 0.057937044 | 1 | 0.246806 | 1 |
| 48 | n294055 | 4042 | 3078.73 | 3136.36 | 3200.87 | 0.916599946 | 1 | 0.873023 | 1 |
| 49 | XLOC_028221 | 7636 | 3036.64 | 3000.19 | 7090.57 | 0.948210216 | 1 | 9.55E-06 | 0.003027 |

| | | | | | | | | | |
|---|---|---|---|---|---|---|---|---|---|
| 50 | n177784 | 7003 | 3035.07 | 2177.29 | 3317.68 | 0.036089707 | 1 | 0.007699 | 0.281619 |
| 51 | n344280 | 8745 | 3009.39 | 2996.07 | 2655.79 | 0.986343549 | 1 | 0.423545 | 1 |
| 52 | n413093 | 8300 | 2931.68 | 2941.73 | 2752.98 | 0.980521362 | 1 | 0.689114 | 1 |
| 53 | XLOC_024241 | 7110 | 2930.58 | 2815.49 | 2704.34 | 0.802012516 | 1 | 0.812803 | 1 |
| 54 | XLOC_062599 | 3313 | 2904.04 | 3158.24 | 2925.90 | 0.579683412 | 1 | 0.621003 | 1 |
| 55 | n416967 | 3632 | 2900.69 | 5801.82 | 3262.28 | 0.000528753 | 0.120491 | 0.003676 | 0.185668 |
| 56 | n281790 | 1616 | 2890.18 | 2568.70 | 1978.51 | 0.670730737 | 1 | 0.339037 | 1 |
| 57 | n273000 | 3165 | 2845.46 | 3110.70 | 3038.41 | 0.570839738 | 1 | 0.902932 | 1 |
| 58 | XLOC_075409 | 547 | 2810.92 | 2175.98 | 2616.59 | 0.395437418 | 1 | 0.560003 | 1 |
| 59 | XLOC_014581 | 213 | 2780.34 | 2139.45 | 2997.28 | 0.359101221 | 1 | 0.252371 | 1 |
| 60 | XLOC_009151 | 2725 | 2764.53 | 3068.84 | 2694.09 | 0.49921767 | 1 | 0.405486 | 1 |
| 61 | n295660 | 1644 | 2745.01 | 2442.50 | 2949.83 | 0.474041129 | 1 | 0.226784 | 1 |
| 62 | XLOC_028567 | 5422 | 2696.55 | 2798.11 | 2460.44 | 0.815761031 | 1 | 0.418415 | 1 |
| 63 | XLOC_071148 | 5345 | 2695.29 | 2642.61 | 2026.13 | 0.912539842 | 1 | 0.087419 | 0.846212 |
| 64 | n276978 | 4874 | 2679.74 | 3575.35 | 2978.49 | 0.063909558 | 1 | 0.236643 | 1 |
| 65 | n270661 | 3827 | 2630.06 | 2466.08 | 1558.94 | 0.673148532 | 1 | 0.004108 | 0.197633 |
| 66 | n270770 | 3837 | 2557.61 | 2369.01 | 1991.96 | 0.634663663 | 1 | 0.265683 | 1 |
| 67 | n284801 | 1857 | 2556.46 | 2298.84 | 2284.49 | 0.497955829 | 1 | 0.987197 | 1 |
| 68 | XLOC_074811 | 5741 | 2532.40 | 2542.64 | 2371.11 | 0.982073734 | 1 | 0.68054 | 1 |
| 69 | n291463 | 1293 | 2493.27 | 2151.59 | 1635.00 | 0.560425479 | 1 | 0.292197 | 1 |
| 70 | XLOC_009164 | 9113 | 2478.15 | 2498.43 | 2919.39 | 0.964706875 | 1 | 0.316021 | 1 |
| 71 | n273724 | 3795 | 2467.43 | 2569.53 | 1896.58 | 0.797839228 | 1 | 0.057596 | 0.732701 |
| 72 | XLOC_024053 | 7080 | 2444.12 | 3115.26 | 2685.06 | 0.131384032 | 1 | 0.361946 | 1 |
| 73 | XLOC_018312 | 5223 | 2419.00 | 2458.02 | 2233.77 | 0.919394164 | 1 | 0.553833 | 1 |
| 74 | n264913 | 6852 | 2380.39 | 2470.00 | 3001.94 | 0.81580392 | 1 | 0.215127 | 1 |
| 75 | n281738 | 1630 | 2375.24 | 1636.92 | 2948.14 | 0.1023855 | 1 | 0.010333 | 0.333961 |

| | | | | | | | | | |
|---|---|---|---|---|---|---|---|---|---|
| 76 | XLOC_044900 | 8081 | 2368.79 | 2517.65 | 2514.87 | 0.6912762 | 1 | 0.996738 | 1 |
| 77 | n282758 | 3487 | 2349.17 | 2124.18 | 1852.85 | 0.535701462 | 1 | 0.389278 | 1 |
| 78 | XLOC_059758 | 7157 | 2347.02 | 2444.47 | 2671.64 | 0.777900887 | 1 | 0.590967 | 1 |
| 79 | n265894 | 2806 | 2321.64 | 2302.42 | 3725.60 | 0.990556648 | 1 | 0.002328 | 0.142357 |
| 80 | XLOC_064946 | 1673 | 2316.66 | 2008.60 | 1851.87 | 0.529443876 | 1 | 0.726575 | 1 |
| 81 | n278853 | 427 | 2313.75 | 2554.03 | 2375.07 | 0.542590951 | 1 | 0.678623 | 1 |
| 82 | XLOC_064989 | 9231 | 2282.65 | 1292.72 | 833.55 | 0.000451304 | 0.107429 | 0.006937 | 0.264336 |
| 83 | n290245 | 1770 | 2251.07 | 2060.12 | 1644.41 | 0.575609663 | 1 | 0.154964 | 1 |
| 84 | XLOC_018256 | 6758 | 2244.47 | 2303.86 | 2656.99 | 0.869628878 | 1 | 0.35901 | 1 |
| 85 | n283249 | 3198 | 2239.33 | 3575.98 | 3495.49 | 0.002946898 | 0.319064 | 0.881755 | 1 |
| 86 | n288273 | 2761 | 2221.00 | 1860.00 | 4476.44 | 0.383263259 | 1 | 8.47E-06 | 0.0028 |
| 87 | n265717 | 2228 | 2187.70 | 2220.26 | 3542.55 | 0.904147224 | 1 | 0.003101 | 0.167415 |
| 88 | n267330 | 3483 | 2171.38 | 1955.99 | 3475.52 | 0.514484019 | 1 | 0.000297 | 0.037448 |
| 89 | n287231 | 1857 | 2162.55 | 2203.14 | 2046.96 | 0.901419847 | 1 | 0.654582 | 1 |
| 90 | n276979 | 2863 | 2155.23 | 1814.63 | 2129.08 | 0.289997339 | 1 | 0.312728 | 1 |
| 91 | n274097 | 3772 | 2148.93 | 2216.29 | 3176.15 | 0.825246282 | 1 | 0.022366 | 0.488431 |
| 92 | XLOC_035064 | 2518 | 2147.44 | 1606.19 | 2290.38 | 0.064581754 | 1 | 0.023387 | 0.499119 |
| 93 | XLOC_058975 | 1911 | 2124.01 | 2712.88 | 2570.59 | 0.399280309 | 1 | 0.830013 | 1 |
| 94 | n286590 | 4974 | 2113.84 | 2353.98 | 1730.11 | 0.515531318 | 1 | 0.055366 | 0.722036 |
| 95 | XLOC_007318 | 5616 | 2109.42 | 2242.53 | 1722.92 | 0.704017067 | 1 | 0.091552 | 0.856264 |
| 96 | n421379 | 797 | 2098.98 | 1881.99 | 1682.73 | 0.508491083 | 1 | 0.476048 | 1 |
| 97 | n286082 | 3012 | 2071.11 | 2574.06 | 2188.90 | 0.169064454 | 1 | 0.307824 | 1 |
| 98 | n288898 | 2850 | 2069.46 | 1868.35 | 1830.63 | 0.521692489 | 1 | 0.899597 | 1 |
| 99 | XLOC_018797 | 2149 | 2067.90 | 1904.23 | 2046.20 | 0.608579703 | 1 | 0.640351 | 1 |
| 100 | XLOC_074207 | 9327 | 2056.46 | 1923.15 | 2569.23 | 0.693372116 | 1 | 0.083500 | 0.832416 |

* ID number was obtained from NONCODE v3.0 database.

**Supplemental Table 2 Oligonucleotide primer sequences**

| Designation | Forward | Reverse |
|---|---|---|
| ***in situ* hybridization** | | |
| XLOC_033862_probe_1 | TGA CTT GAG GCT TTC ATC ATC TGG | CTG TTG TCC TGG CTG TGC TCT TTA |
| **RT-PCR** | | |
| XLOC_033862 | TGC AGC TAA GAA TGA CCC AG | CTT CAA GTT CAT CCC AAC ACA G |
| *Gfra1* | CTC GGA ATC CAG CCT ACG TC | CAC TTG TCC TCT CGT GTG CT |
| *Etv5* | CAC CAT GTA TCG AGA GGG GC | GAG CAA CCT CTT CCG GTT CT |
| *Pou5f1* | TGG AGA AGG TGG AAC CAA CTC CC | ACA CGG TTC TCA ATG CTA GTT CGC |
| *Zbtb16* | CTG CGG AAA ACG GTT CCT G | GTG CCA GTA TGG GTC TGT CT |
| *Bcl6b* | TAC TTC AAG GCT TCG CCT CTC T | CTA CGT GTT CCA TCT GCA AAT AGG |
| *Ccnd2* | CTG CGG AAA AGC TGT GCA TT | AAC TTG AAG TCG GTA GCG CA |
| *Cxcr4* | ATG GAA CCG ATC AGT GTG AG | GAT GAA GTA GAT GGT GGG CAG |
| *kit* | TGT GGC TAA AGA TGA ACC CTC | ACA CTC CAG AAT CGT CAA CTC |
| *β1-integrin* | GCA ACG CAT ATC TGG AAA CTT G | CAA AGT GAA ACC CAG CAT CC |
| *Stra8* | TTA AAC CAG GAA CCA GAG CC | AAG ACA AGG CAG TAT AAC TCT AGC |
| *Sycp1* | AGT CGG GAA AAC ATT GAT AAA GAT C | ATA CAG TCT GCT CAT TGG CTC |

| | | |
|---|---|---|
| *Actin* | CCG TAA AGA CCT CTA TGC C | CTC AGT AAC AGT CCG CCT A |
| **PCR** | | |
| *Pou5f1* | TGG AGA AGG TGG AAC CAA CTC CC | ACA CGG TTC TCA ATG CTA GTT CGC |
| *Lin28a* | AGA TGC TCA AAG AAG TAA ATG | TCC TCT TCT CAA AGC GAA CCT |
| *Sox2* | GCA CAT GAA CGG CTG GAG CAA CG | TGC TGC GAG TAG GAC ATG CTG TAG G |
| *Vasa* | TGA TTC AGG CAA TGG TGA CAC T | TTC CCA GAC CCT GTT TGA GC |
| *Dazl* | CTC TCT TTC CAC CAC CGC C | AGG GCC CAG TTT CAG CTT TT |
| *Gfra1* | CTC GGA ATC CAG CCT ACG TC | CAC TTG TCC TCT CGT GTG CT |
| *Zbtb16* | CTG CGG AAA ACG GTT CCT G | GTG CCA GTA TGG GTC TGT CT |
| *Gata-1* | ATC GCC CCA CTC AGT TCT TG | ATA CCA GCG CAT GTC TCC AG |
| *Actin* | CCG TAA AGA CCT CTA TGC C | CTC AGT AAC AGT CCG CCT A |
| **shRNA** | | |
| XLOC_033862-663 | CCGGGTCCATAGTCTCTGTATTATACTCGAGTATAATACAGAGACT ATGGACTTTTTG | AATTCAAAAAGTCCATAGTCTCTGTATTATACTCGAGTATAA TACAGAGACTATGGAC |
| XLOC_033862-1894 | CCGGAGTAAGGCCTCTGCTATTATTCTCGAGAATAATAGCAGAGG CCTTACTTTTTTG | AATTCAAAAAAGTAAGGCCTCTGCTATTATTCTCGAGAATA ATAGCAGAGGCCTTACT |
| **Cytoplasma and nucleus separation** | | |

| | | |
|---|---|---|
| *U6 snRNA* | CTC GCT TCG GCA GCA CA | AAC GCT TCA CGA ATT TGC GT |
| *Actin* | CCG TAA AGA CCT CTA TGC C | CTC AGT AAC AGT CCG CCT A |
| XLOC_033862 | TGC AGC TAA GAA TGA CCC AG | CTT CAA GTT CAT CCC AAC ACA G |
| **Polysome separation** | | |
| XLOC_033862 | TGC AGC TAA GAA TGA CCC AG | CTT CAA GTT CAT CCC AAC ACA G |
| *U6 snRNA* | CTC GCT TCG GCA GCA CA | AAC GCT TCA CGA ATT TGC GT |
| *GAPDH* | GGT GAA GGT CGG TGT GAA CG | CTC GCT CCT GGA AGA TGG TG |
| **ChIRP** | | |
| *GAPDH* | GGT GAA GGT CGG TGT GAA CG | CTC GCT CCT GGA AGA TGG TG |
| XLOC_033862 | TGC AGC TAA GAA TGA CCC AG | CTT CAA GTT CAT CCC AAC ACA G |
| XLOC033862-1 | GGT CTC CAA GAG ACA TAT CA | |
| XLOC033862-2 | TTA CAA TAC TGA GAG TGC CC | |
| XLOC033862-3 | CAG TCT GGA ACT TTC TTA CC | |
| XLOC033862-4 | CGG ATG CTC ATT TTG TGG AA | |
| XLOC033862-5 | AGT GAC TAA TGG AGG GCT TT | |
| XLOC033862-6 | TCA AGT TCA TCC CAA CAC AG | |
| XLOC033862-7 | GAA TTT GGA ATG CTG GCT GT | |

| | |
|---|---|
| XLOC033862-8 | TAC ATG GTC AGG CTC AGA AT |
| XLOC033862-9 | AGA TCA AGG CTG CCT ATA TG |
| XLOC033862-10 | CCA CAT CTT ACG AAC GTT CA |

260
